# Loss of function of the maternal membrane oestrogen receptor ERα alters expansion of trophoblast cells and impacts mouse fertility

**DOI:** 10.1242/dev.200683

**Published:** 2022-10-13

**Authors:** Mariam Rusidzé, Mélanie C. Faure, Pierre Sicard, Isabelle Raymond-Letron, Frank Giton, Emilie Vessieres, Vincent Prevot, Daniel Henrion, Jean-François Arnal, Charlotte A. Cornil, Françoise Lenfant

**Affiliations:** ^1^ Institute of Metabolic and Cardiovascular Diseases (I2MC) Equipe 4, Inserm U1297-UPS, CHU, Toulouse 31432, France; ^2^ GIGA Neurosciences, University of Liège, Liège 4000, Belgium; ^3^ IPAM, BioCampus Montpellier, CNRS, INSERM, University of Montpellier, Montpellier 34295, France; ^4^ Institut Restore, Université de Toulouse, CNRS U-5070, EFS, ENVT, Inserm U1031, Toulouse 31076, France; ^5^ APHP H.Mondor - IMRB - INSERM U955, Créteil 94010, France; ^6^ Angers University, MITOVASC, CarMe team, CNRS UMR 6015, INSERM U1083, Angers 49055, France; ^7^ University of Lille, Inserm, CHU Lille, Laboratory of Development and Plasticity of the Neuroendocrine Brain, Lille Neuroscience and Cognition, UMR-S 1172, FHU 1000 Days for Health, Lille 59000, France

**Keywords:** Membrane signalling, Oestrogen receptor ERα (ESR1), Fertility, Trophoblast cells, Spiral arterial remodelling, Parturition

## Abstract

The binding of 17β-oestradiol to oestrogen receptor alpha (ERα) plays a crucial role in the control of reproduction, acting through both nuclear and membrane-initiated signalling. To study the physiological role of membrane ERα in the reproductive system, we used the C451A-ERα mouse model with selective loss of function of membrane ERα. Despite C451A-ERα mice being described as sterile, daily weighing and ultrasound imaging revealed that homozygous females do become pregnant, allowing the investigation of the role of ERα during pregnancy for the first time. All neonatal deaths of the mutant offspring mice resulted from delayed parturition associated with failure in pre-term progesterone withdrawal. Moreover, pregnant C451A-ERα females exhibited partial intrauterine embryo arrest at about E9.5. The observed embryonic lethality resulted from altered expansion of *Tpbpa*-positive spiral artery-associated trophoblast giant cells into the utero-placental unit, which is associated with an imbalance in expression of angiogenic factors. Together, these processes control the trophoblast-mediated spiral arterial remodelling. Hence, loss of membrane ERα within maternal tissues clearly alters the activity of invasive trophoblast cells during placentogenesis. This previously unreported function of membrane ERα could open new avenues towards a better understanding of human pregnancy-associated pathologies.

## Teaser

**Summary:** A point mutation in the oestrogen receptor ERα alters pregnancy, revealing the crucial role for this receptor in maternal tissues for both placental development and delivery.

## INTRODUCTION

Oestrogens exert a crucial role in fertility, including reproductive organogenesis, sexual behaviour and ovulation. In many species, ovarian 17β-oestradiol (E2) and locally synthesized placental oestrogens act in concert to mediate their actions through two main receptors: ERα (also known as ESR1) and ERβ (ESR2) (Malassine et al., 2003; Das et al., 2009; Mesiano, 2019). Both receptors are required for reproduction (Dupont et al., 2000; Antal et al., 2008; Hewitt and Korach, 2018). However, ERα appears as the key mediator in fertility through its multiple actions on reproductive tissues. The infertility of ERαKO mice, which is characterized by anovulation, acyclicity and uterine atrophy, is explained by both central and peripheral defects (Dupont et al., 2000). ERα elicits transcriptional activation of downstream target genes through genomic and non-genomic signalling pathways (Arnal et al., 2017). Many experiments provided compelling evidence of genomic/nuclear actions of ERα in the reproductive system through AF1 and AF2 transactivation functions. Knock-in mouse models with deletions for either AF1 or AF2 function exhibit polycystic ovaries and atrophic uteri that entirely prevent the embryonic implantation that is necessary for a successful pregnancy (Billon-Galés et al., 2009, 2011).

Besides these genomic actions, a few studies suggested a role for the non-genomic signalling pathway of ERα during fertility. These non-genomic pathways activate very rapid signalling (from seconds to few minutes), such as an increase in cAMP in the uterus in response to E2 (Szego and Davis, 1967) and the activation of several kinase cascades (Arnal et al., 2017). Two similar knock-in mouse models selectively inactivated for membrane functions of ERα have been generated by mutating the cysteine 451 into an alanine (447 counterpart in humans), which impairs ERα palmitoylation and then membrane localization. Both C451A-ERα (Adlanmerini et al., 2014) and nuclear-only ERα (NOER) mice (Pedram et al., 2014) show abnormal ovaries with haemorrhagic cysts and no corpus luteum, suggesting anovulation, whereas the uterine response to E2 varies from normal in C451A-ERα (Adlanmerini et al., 2014) to a 40 to 100% reduction in size in NOER mice (Pedram et al., 2014; Gustafsson et al., 2016). Moreover, rapid vascular effects of E2, such as vasodilatation, acceleration of endothelial repair and endothelial NO synthase phosphorylation, were abrogated in C451A-ERα mice (Adlanmerini et al., 2014). Both models exhibit several reproductive abnormalities and were described as infertile because of the absence of offspring, despite repeated mating (Adlanmerini et al., 2014; Pedram et al., 2014). However, the mechanisms involved in the loss of reproductive function remained to be explored. In this context, we performed an in-depth study of C451A mice infertility. Unexpectedly, these mice could initiate a pregnancy, although they displayed several gestational and parturition abnormalities, leading to final neonatal lethality of offspring. Hence, total litter loss explains previously reported data.

The principal roles of oestrogens in placentation are the stimulation of uterine vascular growth through the secretion of angiogenic factors and the regulation of the uterine blood flow via nitric oxide (NO)-dependent and -independent manners (Chang and Zhang, 2008; Corcoran et al., 2014). However, although the regulatory role of oestrogens on decidualization, trophoblast viability, differentiation, proliferation and invasiveness is evidenced in human and non-human primates, little is known about the roles of ERα in the murine trophoblast biology (Bukovsky et al., 2003; Albrecht et al., 2006; Kumar et al., 2009; Maliqueo et al., 2016; He et al., 2019). In addition to these effects, labour onset in mice also depends on oestradiol, the circulating levels of which remarkably rise before parturition in parallel to progesterone withdrawal, altogether allowing uterine contractility and labour initiation (Kota et al., 2013).

Although almost all knockout and knock-in mouse models for ERα are infertile (Dupont et al., 2000; Billon-Galés et al., 2009, 2011; Adlanmerini et al., 2020), the C451A-ERα model is the only one allowing the study of maternal ERα actions throughout pregnancy. Our main results reveal placental developmental abnormalities of C451A-ERα mice, associated with the dysregulation of the specific markers of spiral arterial-associated trophoblast giant cells (SpA-TGCs) and angiogenic factors at E9.5. Immunodetection of these Tbpba-positive specialized trophoblast cells confirmed their reduced expansion through the utero-placental unit of C451A-ERα mothers compared with their wild-type littermates. Nonetheless, these invading trophoblast cells were absent in dying embryos, highlighting the role of maternal membrane ERα signalling in the control of trophoblast biology and therefore for pregnancy outcome.

## RESULTS

### C451A-ERα females do become pregnant but exhibit delayed parturition that results in total neonatal mortality of pups

**Fig. 1.**
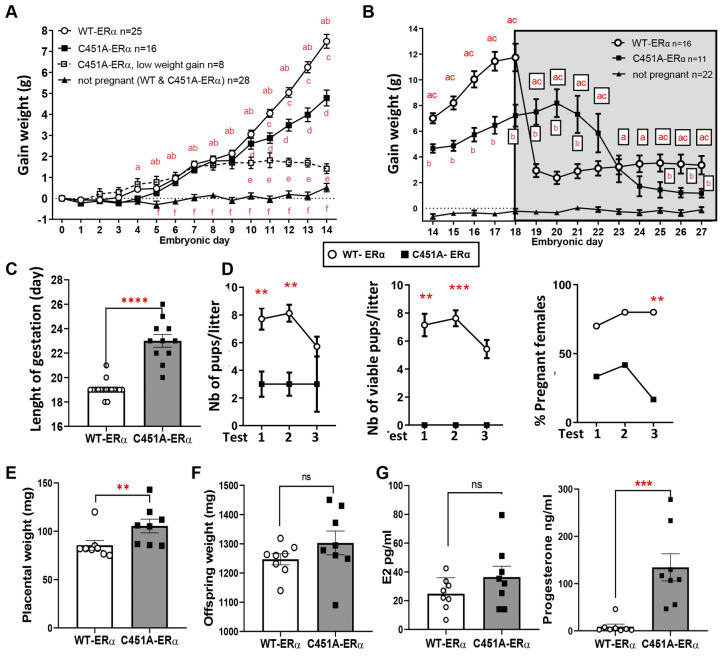
**C451A-ERα females can become pregnant but exhibit parturition failure that results in absence of offspring.** (A,B) Evolution of body weight gain from embryonic day (E) 0.5 to E14.5 (A) and E14.5 to E27.5 (B) in WT-ERα and C451A-ERα females, mated with WT-ERα males. Two-way ANOVA with Tukey's post-hoc test. ^a^*P*<0.05 for WT-ERα versus not pregnant; ^b^*P*<0.05 for C451A-ERα versus not pregnant; ^c^*P*<0.05 for WT-ERα versus C451A-ERα; ^d^*P*<0.05 for C451A-ERα versus C451A-ERα with low weight gain; ^e^*P*<0.05 for WT-ERα versus C451A-ERα with low weight gain; ^f^*P*<0.05 for C451A-ERα with low weight gain versus not pregnant. Mice were considered to be pregnant if they had gained more than 1 g by E7 or as non-pregnant if they had gained less than 1 g by E7. However, if they had gained more than 1 g by E7 but their weight gain had not doubled by E14, mice were classified as low weight gain. Resorptions were detected in the uteri of two mice sacrificed at E14, demonstrating that they could not be considered to be pregnant nor non-pregnant. (C) Gestational lengths of pregnant WT-ERα and C451A-ERα females that gave birth between E18.5 and E26.5. ****P<0.0001 (Mann–Whitney test). (D) Number of total (left) and live (middle) pups per litter found in nests on postnatal day 1 and percentage of pregnant females (right) after three consecutive mating tests in WT-ERα (*n*=10) and C451A-ERα mice (*n*=12), mated with WT-ERα male for 7 days. Mann–Whitney tests or Fisher's exact test: **P<0.01 and ***P<0.001. Data are representative of three independent experiments. (E,F) Placental (E) and offspring body (F) weights of foetuses from C451A-ERα mice (*n*=8) and WT-ERα littermates (*n*=8) at E18.5 after caesarean sections. **P<0.01 (Mann–Whitney test). Each point corresponds to the means of all *concepti* per mother. (G) Circulating levels of 17β-oestradiol (E2) and progesterone in pregnant WT-ERα (*n*=8) and C451A-ERα (*n*=8) mice at E18.5. ***P<0.001 (Mann–Whitney test).

An exhaustive study was performed to gain insights into the causes of the infertility of the C451A-ERα mice. Females were mated overnight with wild-type ERα (WT-ERα) males and body weight gain was monitored daily starting at E0.5, as defined by vaginal plug detection. We found that a large number of females of both genotypes gradually gained weight from E0.5 to E7.5, suggesting they were pregnant, whereas others exhibited no weight gain and were therefore considered non-pregnant (Fig. 1A). This was later confirmed by the observation of presence/absence of foetuses or pups in the womb or nests, respectively. Interestingly, starting at E10.5, the weight gain profile of C451A-ERα mice started to diverge from WT-ERα females. One subset of C451A-ERα females (low weight gain, *n*=8) clearly stopped gaining weight, possibly because of abortion, whereas the others kept gaining weight but to a lesser extent than WT-ERα females. This lower gestational weight gain in C451A-ERα mice compared with wild type became statistically significant at E11.5 and was maintained until E18.5 (Fig. 1B). At this time, WT-ERα females exhibited a dramatic weight drop accompanied by the delivery of an average of seven or eight viable pups (Fig. 1B). In contrast, C451A-ERα mice demonstrated a progressive reduction in average body weight between E20.5 and E27.5, which is indicative of delayed parturition (Fig. 1B,C). Moreover, the analysis of gestational success after three successive mating sessions with WT-ERα males indicated that C451A-ERα mice had significantly fewer pups and no live offspring were observed within 24 h of parturition (Fig. 1D). These pups were already dead at the moment of parturition or died before birth, although they did not present overt malformations (Fig. S1). Overall, fewer C451A-ERα females were pregnant over three successive mating sessions compared with WT-ERα females (Fig. 1D). Thus, C451A-ERα females can become pregnant but exhibit parturition failure and total neonatal mortality of pups.

Although parturition was largely delayed to around day E22.5 in C451A-ERα mice, the intrauterine viability of pups was first evaluated at E18.5 following caesarean sections to investigate the causes of postnatal lethality. The foetuses delivered from C451A-ERα females by caesarean sections were still viable, as defined by the presence of a heartbeat with no apparent congenital anomalies. However, placental weight was increased in embryos of C451A-ERα females compared with WT-ERα females (Fig. 1E), although offspring weights were not significantly affected (Fig. 1F). Analysis of circulating sex steroid levels before expected parturition (E18.5) revealed 16-fold higher circulating progesterone levels in C451-ERα mice at E18.5, compared with WT-ERα littermates, suggesting a failure in the pre-partum decline of progesterone in mutant females (Fig. 1G). By contrast, circulating oestradiol (E2) levels were similar in both WT-ERα and C451-ERα females before parturition (E18.5; Fig. 1G), thus highlighting unaltered pre-partum E2 production in C451-ERα mice. Other circulating steroid levels at term were unchanged, except for androsterone and dehydroepiandrosterone (DHEA) (Table S1). Altogether, these data indicate that C451A-ERα females do become pregnant, but demonstrate aberrant parturition and subsequent offspring loss, likely due to the failure of progesterone withdrawal at the end of gestation.

### C451A-ERα mothers exhibit partial intrauterine lethality at mid-gestation

**Fig. 2.**
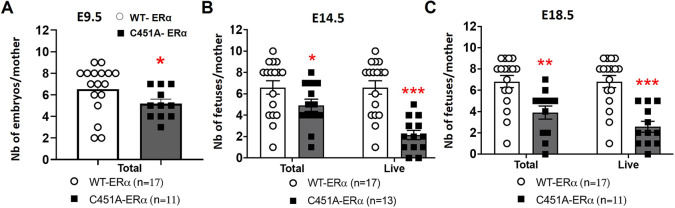
**ERα-C451A mice exhibit intra-uterine embryonic lethality.** (A-C) Number of total (A), and total and live (B,C) embryos per mother at E9.5 (A), E14.5 (B) and E18.5 (C), evidenced by ultrasound imaging. *P<0.05, **P<0.01 and ***P<0.001 (Mann–Whitney tests). Dots on the graphs represent numbers from a single mother.

Ultrasound imaging was then applied to follow the embryonic prenatal development and investigate the potential pregnancy-associated complications in mutant mice (Fig. 2). We confirmed that C451A-ERα mothers had significantly fewer implantation sites at E9.5 than their wild-type littermates (Fig. 2A). Furthermore, mutant females carried even fewer live embryos at E14.5 than E9.5, an observation indicative of miscarriages (Fig. 2B). Notably, most live embryos identified at E14.5 remained alive until term (E18.5) (Fig. 2C), excluding additional abortions after E14.5.

**Fig. 3.**
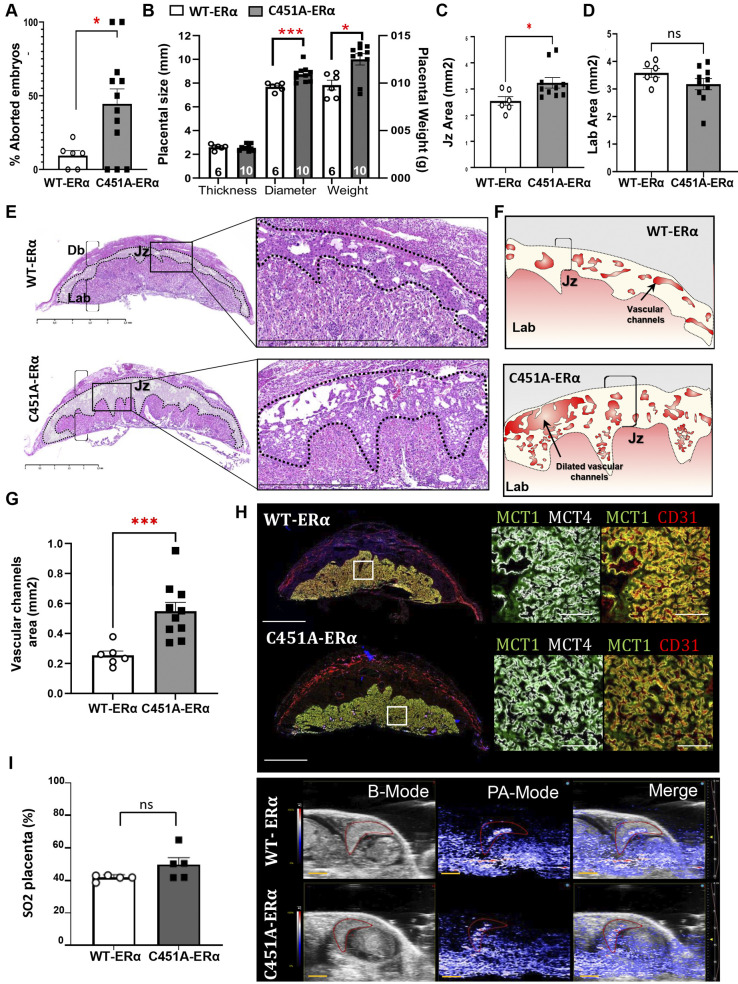
**C451A-ERα pregnant females show placentomegaly due to dilated vascular channels in the junctional zone.** (A) Percentages of aborted embryos observed in pregnant C451A-ERα (*n*=6) and WT-ERα (*n*=12) mice. *P<0.05, Mann–Whitney test. (B) Placenta thickness, diameter and weight at E14.5. *P<0.05, ***P<0.001, Mann–Whitney test. (C,D) Quantifications of junctional zone (C) and labyrinth (D) areas (mm^2^). Data include 32 placentas from 14 mothers (*n* for WT-ERα=6 and *n* for C451A-ERα=10). Dots represent the mean areas of all placentas obtained from the same mother. *P<0.05, Mann–Whitney test. (E) Transverse histological sections of whole placentas (left) collected from WT-ERα and C451A-ERα mice at E14.5 showing all major placental structural components. Magnified views of the junctional zone (Jz), delineated by a dotted line, are shown in the right inset. Db, decidua basalis; Jz, junctional zone; Lab, labyrinth. Scale bars: 2.5 mm (right) and 1 mm (left). (F,G) Respective quantification (G) with schematic illustration (F) of the surface of vascular channels (red area) in the junctional zone (dotted line) represented by average measurements per mother. The data highlight the dilated vascular tree of Jz in placentas of C451A-ERα mice (right, *n*=10), compared with the WT-ERα mice (left, *n*=6). ***P<0.001 Mann–Whitney test. (H) Double staining of MCT1 (green) and MCT4 (red) or MCT1 (green) and CD31 (red) mouse placental sections at E14.5. Labelling of maternal vasculature (CD31) and of apical (MCT1) and basal (MCT4) plasma membranes of labyrinthine syncytiotrophoblast cells. Scale bars: 2 mm (left); 100 μm (right). (I) Oxygen saturation values (SO2) of whole placental tissue at E14.5, represented by average measurements per mother. SO2 was measured in two to five live embryos per mother. WT-ERα (*n*=5) and C451A-ERα (*n*=5). Scale bars: 3 mm. (J) Examples of B-mode and photoacoustic-mode (PA) *in vivo* images of SO2 measurements at E14.5. The merge panel illustrates the custom colour map of oxygen saturation superimposed on the ultrasound image, where red corresponds to completely oxygenated blood and blue to completely deoxygenated blood. Scale bar: 2 mm.

Post-mortem analyses of C451A-ERα females also detected fewer live embryos and higher percentages of aborted embryos than wild-type littermates at E14.5, confirming ultrasound findings (Fig. 3A,B; Fig. S2A,B). Surviving embryos of WT-ERα and C451A-ERα mothers lacked overt developmental abnormalities and presented similar crown-rump lengths and body weights, suggesting an absence of growth retardation (Fig. S2C).

To pinpoint the causes of reduced implantation sites in mutant females, whether C451A-ERα mice display some ovulatory dysfunction was investigated. The number of spontaneously ovulated oocytes was quantified in 3-month-old females, bearing a vaginal plug on E0.5 after mating with WT-ERα males. There was no difference between genotypes in the percentage of females with plug (WT-ERα, 21.02±6.55; C451A-ERα, 21.01±10.81), in the percentage of females that ovulated (83% of WT and 93% C451A-ERα females) and in the number of ovulated oocytes (Fig. S3). Therefore, the difference in live embryos reported here does not seem to stem from a difference in ovulation after mating with wild-type males.

### C451A ERα mice show placentomegaly due to dilated vascular channels in the junctional zone

Although we did not detect any growth retardation in the surviving embryos at E14.5, the placental weights and diameters of C451A-ERα females were significantly increased, compared with those of wild-type controls (Fig. 3B). The nature of placentomegaly was further explored by histological analyses of placental middle sections at E14.5. Both maternal and foetal-derived structures, including decidua basalis (Db), junctional zone (Jz) and placental labyrinth layer (Lab) were present and no major structural defects, such as necrosis or haemorrhages, were observed in placentas from mothers of both genotypes (Fig. 3C-E). Although the surface of the labyrinth layer did not differ between genotypes, the area of the Jz was significantly increased in mutant mouse placentas, compared with their wild-type littermates (Fig. 3C,D). Interestingly, a selective increase in the vascular channel area of the junctional zone was observed (Fig. 3F,G).

To define more precisely the structure of the placental vascular tree, immunofluorescence co-labelling was performed on the maternal endothelium (CD31) and in the labyrinthine syncytiotrophoblasts, which express monocarboxylate transporter (MCT) 1 and MCT4 proteins in the apical and basal plasma membranes, respectively (Fig. 3H). No major abnormalities of maternal uterine vessels or of the fine labyrinthine vascular network were observed. Additionally, photoacoustic imaging (PAI) was used to assess the functional capacity of surviving embryo placentas. This non-invasive technique allowed us to measure the *in vivo* real-time oxygen uploading process in placental tissues (SO2), based on the difference between the average concentrations of placental oxyhaemoglobin (HbO2) and placental deoxyhaemoglobin (HB) (Fig. 3I, left panel) (Wang et al., 2016). According to the unaltered vascular structure of survival embryo placentas, the oxygen saturation (SO2) in E14.5 placentas did not differ between genotypes (Fig. 3I). Altogether, these data show that the placentas of surviving embryos of C451A-ERα mice display an increased junctional zone with dilated vascular channels compared with those of wild-type controls, without alteration of placental perfusion and its oxygen supplying functions.

### The physiological remodelling of uterine arteries is preserved in C451A-ERα pregnant mice

Defective development of placental vasculature is one, if not the most, frequent causes of pregnancy-associated complications in humans and rodents (Berkane et al., 2017). Based on the profile of gestational weight gain and post-mortem analyses, the time of embryo abortion in C451A-ERα females was estimated to be between E9.5 and E13.5, when the utero-placental circulation starts in mice (Georgiades et al., 2002). Given the modified placental vasculature observed in mutant mice, we further investigated two hallmark vascular events that precede this time point of pregnancy and are highly regulated by oestrogens: physiological remodelling of uterine arteries and spiral arterial remodelling (Mandala, 2020).

**Fig. 4.**
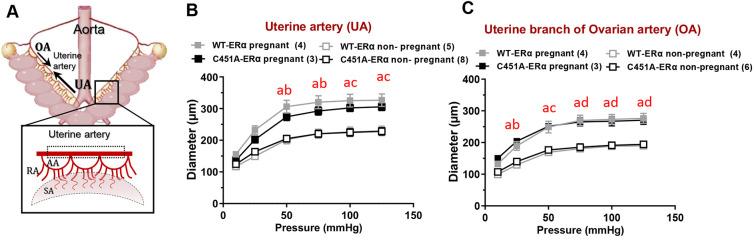
**Physiological remodelling of resistance uterine arteries is not affected by membrane ERα loss of function.** (A) Organization of the uterine circulation in the pregnant mouse. Mouse pregnancy is supported by two uterine arteries, one on each side of the uterine horn (left and right). This arterial loop is generated from direct anastomoses of the uterine branch of the ovarian artery (OA) (cranially) and the uterine artery (UA) (caudally), resulting in counter bi-directional blood flow. The uterine artery further branches out to the arcuate artery (AA), the radial artery (RA) and the spiral artery (SA), which, respectively, supply the endometrium, decidua and placenta during pregnancy. Dotted rectangle outlines the uterine arterial segment that was used for this experiment. (B,C) Diameter (µm) of two fragments (UA and OA) of uterine arteries isolated from non-pregnant and pregnant WT-ERα and C451A-ERα mice at E9.5 after a stepwise increase in intraluminal pressure. Data show a significant pressure effect in pregnant and non-pregnant females in both genotypes. ^a^*P*<0.0001 versus non-pregnant for wild-type mice; ^b^*P*<0.01, ^c^*P*<0.001 or ^d^*P*<0.0001 versus non-pregnant for C451A-ERα mice following significant interaction in a two-way repeated measure ANOVA. Data are mean±s.e.m.

Pregnancy-associated remodelling of uterine arteries mainly consists of an increase in vascular diameter and blood flow to support the higher nutrient requirement of the growing foetus (Mandala and Osol, 2012). In most systemic vessels, outward remodelling in response to increased flow largely depends on the production of nitric oxide (NO) by endothelial cells. As the rapid NO production is regulated by membrane actions of ERα (Adlanmerini et al., 2014), whether this uterine vascular remodelling was impaired in C451A-ERα mice was investigated. The uterus exhibits a double vascularization by directly anastomosing the uterine artery (UA) and the uterine branch of the ovarian artery (OA) (Fig. 4A). Therefore, both arterial segments were separately subjected to *ex vivo* assessment of uterine arterial remodelling. Progressive increase of internal arterial diameter was then recorded in response to the stepwise elevation of pressure. As expected, the diameters of both segments of the uterine artery were significantly higher in pregnant mice (E9.5) than in the non-pregnant females (Fig. 4B,C). However, no difference was observed between genotypes, indicating that the uterine arterial remodelling and, therefore, the uterine blood supply to downstream spiral arteries was not affected in C451A-ERα mice.

### Membrane loss-of-function of ERα results in reduced expansion of *Tpbpa*-positive spiral artery-associated trophoblast giant cells

Spiral arterial remodelling (SAR) is another crucial event in pregnancy, occurring from about E8.5 in mice and allowing the structural and functional linking of uterine and placental circulations (Adamson et al., 2002; Pijnenborg et al., 2006; Hemberger et al., 2020). A pivotal determinant of SAR is the infiltration of spiral arteries by foetal-derived specialized trophoblast cells, called the spiral artery-associated trophoblast giant cells (SpA-TGCs). These *Tpbpa*-positive cells degrade arterial smooth muscle cells and replace the vascular endothelial lining, thereby transforming pre-existing maternal spiral arteries into low-resistance highly dilated vessels that allow maternal blood to flow into the placenta (Rai and Cross, 2014; Silva and Serakides, 2016). Importantly, ablation of *Tpbpa*-positive precursors of SpA-TGCs results in defective remodelling of maternal spiral arteries and leads to arrest of embryonic development (Hu and Cross, 2011).

**Fig. 5.**
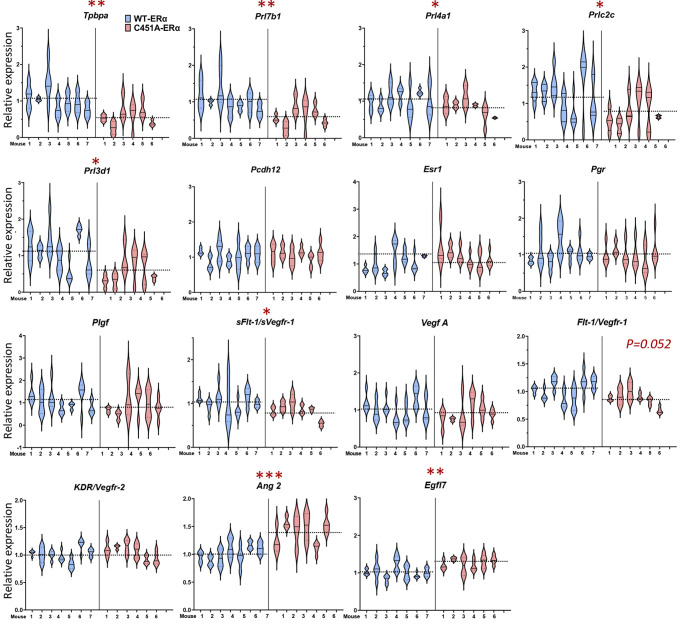
**C451A-ERα mutation in maternal tissues results in dysregulation of genes associated with spiral arterial remodelling in mice.** Real time RT-qPCR for *Tpbpa*, *Prl7b1*, *Prl4a1*, *Prlc2c*, *Prl3d1*, *Pcdh12*, *Esr1*, *Pgr*, *Plgf*, *sFlt1/Vegfr-1*, *Vegf-a*, *Flt1/Vegfr-1*, *KDR/Vegfr-2*, *Ang2* and *Egfl7* genes in whole implantation sites from WT-ERα and C451A-ERα mice (isolated at E9.5). Data include 59 samples, collected from seven WT-ERα and six C451A-ERα mice. Each violin plot represents a distribution of data corresponding to three to five samples obtained from the same mother. Nested *t*-test: *P<0.05, **P<0.01, ***P<0.001. Data are mean±s.e.m.

Subsequently RT-qPCR was performed on all E9.5 *concepti* of mothers of each genotype to profile the gene expression signatures associated with this complex process. Characterization of trophoblast subtypes, which specifically express different subsets of prolactin family member proteins (Simmons et al., 2008; Gasperowicz et al., 2013; Nelson et al., 2016), revealed remarkably reduced expression of several markers specific for the SpA-TGCs (Fig. 5) in the mutant mice *concepti*. These downregulated genes included the trophoblast progenitor marker *Tpbpa,* and also the family prolactin genes *Prl7b1*, *Prl4a1*, *Prlc2c* and *Prl3d1*. Additionally, expression of *Prl3d1,* a gene*-*specific marker of parietal TGCs (P-TGCs), was also decreased in samples of mutant mice. In contrast, the *Pcdh12* gene-specific marker of another *Tpbpa*-positive trophoblastic population, i.e. the glycogen trophoblasts (GlyT), was unchanged in mutant mice *concepti*.

**Fig. 6.**
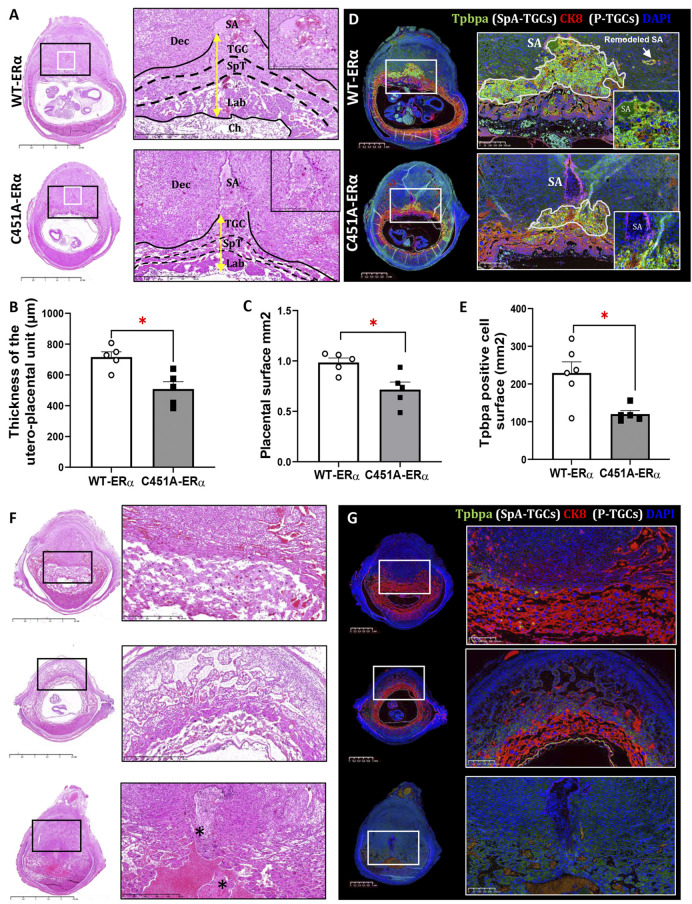
**Histological analysis of *concepti* at E9.5 of WT-ERα and C451A-ERα pregnant females.** (A-C) Histological images of middle sections of whole *concepti* at E9.5, including magnification on the left (A), and respective analyses of the placental surface (mm^2^) (B) and of the thickness of utero-placental unit (C) in C451A-ERα mice (*n*=5) compared with WT-ERα control (*n*=5). Dots represent the mean areas of three or four placentas per mother. SA, spiral artery; TGC, trophoblast giant cell; SpT, spongiotrophoblast; Lab, labyrinth; Ch, chorion. Yellow double-headed arrow indicates the thickness of the utero-placental unit. Scale bars: 2.5 mm, 500 µm and 250 µm (lowest to highest magnifications). *P<0.05, Mann–Whitney test. (D,E) Representative images of the trophoblast progenitor specific marker Tpbpa (green) and CK8 (red) immunostaining in serial sections of whole *concepti* at E9.5 of WT-ERα and C451A-ERα females. Lower and higher magnifications of the area outlined on the left are shown on the right with respective quantification of the cellular surface (mm^2^) positively immunoreactive for anti-Tpbpa antibody (E). Scale bars: 1 mm for images of *concepti*, 250 µm and 100 µm (higher and lower magnification, respectively). *P<0.05, Mann–Whitney test. (F) Representative images of Haematoxylin and Eosin staining of middle sections from some *concepti* of C451A-ERα females at E9.5 that display important disorganization of utero-placental unit showing growth arrest, accompanied by vascular dilatation, hyperaemia, oedema and even total embryo necrosis (asterisks). Scale bars: 2.5 mm. (G) Representative images of the trophoblast progenitor-specific marker Tpbpa (green) and CK8 (red) immunostaining in serial sections of *concepti* at E9.5 of C451A-ERα females where immunostaining of Tpbpa is totally absent. Scale bars: 1 mm (images of *concepti*); 250 μm (higher magnification images).

To further characterize the structural changes associated with these trophoblastic markers, we next performed histological analyses of whole implantations at E9.5. The placental surface was significantly reduced in the C451A-ERα females, compared with their littermate wild-type controls (Fig. 6A,B). Importantly, the thickness of the utero-placental unit, comprising invading TGCs, was also reduced in these placental samples (Fig. 6C). Further immunohistochemical analyses with anti-Tbpba and cytokeratin 8 (CK8) antibodies finally confirmed the considerable decrease in *Tpbpa*-expressing TGCs surface in the utero-placental units of mutant females and highlighted their reduced infiltration of maternal spiral arteries (Fig. 6D,E). Histological examination at E9.5 additionally revealed some dying embryos of C451A-ERα females that demonstrated significant structural and vascular disorganization of the utero-placental unit, which were associated with hyperaemia and oedema, and, occasionally, already apparent embryo necrosis (Fig. 6F). Importantly, *Tpbpa*-expressing TGCs were absent in the placentas of dying embryos (Fig. 6G). Altogether, these data demonstrate that embryo arrest in C451A-ERα females occurs at about E9.5, when the utero-placental vascular circulation begins as a result of the altered expansion of *Tpbpa*-positive SpA-TGCs that control the spiral arterial remodelling. Thus, the membrane function of ERα in maternal cells is required for the SpA-TGCs expansion through the maternal vascular unit and therefore for the control of spiral arterial remodelling.

Because angiogenic factors are well-known regulators of spiral arterial remodelling (Cross et al., 2002) and as their secretion is widely controlled by oestrogens (Albrecht et al., 2004; Das et al., 2009; Albrecht and Pepe, 2010), their relative expression in mouse *concepti* was investigated. First, the expression of *Esr1* (encoding oestrogen receptor ERα) and *Pgr* (encoding progesterone receptor) between C451A-ERα and WT-ERα female *concepti* was unaltered (Fig. 5). Second, significantly reduced expression of soluble vascular endothelial growth factor receptor 1, *sFlt1* (*sVegfr-1*), was observed, whereas expression of major angiogenic genes, such as vascular endothelial vascular growth factor (*Vegfa*), vascular endothelial growth factor receptor 1 (*Flt1*/*Vegfr-1*) and placental growth factor *Pgf*, along with vascular endothelial growth factor receptor 2 (*Kdr*/*Vegfr-2*), did not differ between WT-ERα and C451A-ERa female *concepti* (Fig. 5). By contrast, *Ang2* and the epidermal growth factor like domain 7 (*Egfl7*), which are expressed throughout placental development by both maternal and foetal endothelium, were significantly more expressed in C451A-ERα samples compared with wild-type controls (Lacko et al., 2017). Altogether, this study demonstrated dysregulated expression of some pro- and anti-angiogenic factors in the *concepti* of mutant females.

Finally, expression of Notch gene family members was examined for their importance in the placental vascular development and spiral arterial remodelling (Roca and Adams, 2007; Hunkapiller et al., 2011). We found that the expression of *Notch1* and *Notch4*, one of its ligands, *Dll4*, and its transcription factor *Hey2* was significantly higher in C451A-ERα female *concepti*, whereas another transcription factor, *Hey1*, was downregulated compared with WT-ERα mice (Fig. S4). Altogether, these data suggest that oestrogen-mediated membrane functions of ERα are essential in maternal cells for the regulation of fine-tuned molecular mechanisms implicated in spiral arterial remodelling.

## DISCUSSION

This study reports the first *in vivo* mouse model allowing the investigation of the role of maternal ERα throughout pregnancy. The C451A-ERα model, with membrane loss of function of ERα, presents various pregnancy-associated abnormalities, such as a partial intra-uterine embryonic lethality and parturition failure, that altogether lead to complete neonatal death of offspring. Both abnormalities explain the previously reported infertility of C451A-ERα mice (Adlanmerini et al., 2014). This work provides evidence that maternal ERα controls many functions during placental development and delivery.

Primarily, intrauterine embryonic lethality throughout pregnancy in C451A-ERα females is a crucial finding of this study. Notwithstanding that embryos of C451A-ERα mice were heterozygous for the mutation, the difference in the genotype of embryos between wild-type and C451A-ERα females cannot explain the difference in lethality as all embryos of C451A-ERα females have the same genotype. Moreover, heterozygous females bred with heterozygous males give birth to all genotypes (wild type, heterozygous or homozygous for the mutation) in expected proportions (1, 2 or 1, respectively) following mendelian laws. The similar number of spontaneously ovulated oocytes in C451A-ERα and WT-ERα females following mating with WT-ERα males also ruled out ovulation deficiency as an explanation for the reduced number of implantations in mutant mice. Therefore, this embryonic lethality must result from an alteration of the maternal signals (probably from uterus) for placental development in the ERα-C451A mice.

The characterization of gene expression patterns of different subsets of trophoblast populations (Simmons et al., 2008) revealed a reduced expression of the specific markers associated with two trophoblast lineages at E9.5 in C451A-ERα female *concepti*: SpA-TGCs and P-TGCs. By contrast, the marker associated with glycogen trophoblast cells (Gly-Ts) lineage was unchanged. Interestingly, the SpA-TGCs differentiating from the *Tpbpa*-positive progenitor cells invade and remodel highly resistant maternal spiral arteries into dilated, low-resistant vascular canals close to the P-TGC layer, thereby building connections between maternal and placental circulations (Albrecht et al., 2006; Maliqueo et al., 2016). P-TGCs create extreme edges of the placental periphery and regulate maternal vasculature by facilitating the diffusion of angiogenic and vasoactive substances secreted by different TGCs (Adamson et al., 2002; Cross et al., 2002). Invasion of SpA-TGCs is a pivotal determinant of placentation as the ablation of *Tpbpa*-positive trophoblast cell precursors leads to defective remodelling of spiral arteries and results in embryonic lethality (Hu and Cross, 2011). This dysregulation of gene expression was confirmed by immunohistochemical staining of *Tpbpa*-positive progenitor trophoblast cells, which indicated their altered expansion through the utero-placental unit in the mutant *concepti* at E9.5. This was associated with reduced placental surface and decreased thickness of the utero-placental unit. Reduction of the *Tpbpa*-positive trophoblasts thus explained the intrauterine lethality occurring between E9.5 and E14.5. This is further attested by substantial structural and vascular disorganization, and the absence of *Tpbpa*-expressing TGCs in the placentas of dying embryos of mutant mice using immunostaining. In human beings, disturbed remodelling of maternal uterine spiral arteries leads to increased utero-vascular resistance and is commonly associated with intrauterine growth retardation and pre-eclampsia (Goldman-Wohl and Yagel, 2002; Kaufmann et al., 2003; Young et al., 2010). Embryos from C45A-ERα mothers arrest their development at about E9.5 when the utero-placental circulation becomes functional. This altered expansion of SpA-TGCs is crucial for the spiral arterial remodelling and blood supply. Altogether, these data demonstrate that the membrane function of ERα in maternal tissues is required for the SpA-TGC expansion through the maternal vascular unit and, hence, for the control of spiral arterial remodelling.

The utero-placental vascular development primarily depends on the oestrogen-mediated physiological remodelling of upstream maternal uterine vasculature. This process corresponds to the adaptation of arterial diameter and its wall composition to the increased requirement of blood supply in different physiological conditions (Mandala and Osol, 2012). Membrane ERα was initially reported to be essential for NO-dependent flow-mediated dilation of mesenteric arteries (Adlanmerini et al., 2014). However, *ex vivo* assessment of uterine arterial dilation in response to the applied pressure did not demonstrate any alteration of pregnancy-associated uterine arterial remodelling in C45A-ERα mice at E9.5, thereby suggesting a normal maternal blood supply to the downstream spiral arteries. In fact, these data confirm our previously reported observations in mesenteric arteries of C451A-ERα mice, where the arterial remodelling was entirely preserved 2 weeks after adjacent arterial ligation *in vivo*, although the rapid regulation of NO production was reported to be membrane dependent (Adlanmerini et al., 2014; Guivarc'h et al., 2018). Thus, the combination of these findings strongly indicates that membrane-initiated ERα signalling is not required for the arteriolar remodelling either in mesenteric arteries after ligation or in uterine arteries in response to physiologically increased blood flow during pregnancy.

We also assessed the expression of several pro- and anti-angiogenic genes. C451A-ERα mother *concepti* show decreased expression of *sFlt1*, and high levels of *Ang2* compared with their wild-type littermates. The decreased number of placental cells that secrete this anti-angiogenic factor could be accountable for the decreased expression of *sFlt1* (He et al., 1999; Adamson et al., 2002; Cross et al., 2002). In contrast, Ang2, which is important for placental blood vessel phenotype, was upregulated and high levels of Ang2 have been shown to lead to vascular leakiness with perivascular oedema (Geva et al., 2005). This might contribute to the later changes observed at E14.5. The data also show upregulated expression of *Egfl7* and some of its interacting Notch receptors in C451A-ERα compared with wild-type mice, in particular *Notch 1* and *Notch 4*, and *Hey1* and *Hey2* (downstream mediators of Notch signalling). Oestrogens have been shown to mediate many vascular effects via Notch activation (Soares et al., 2004). Furthermore, Notch signalling has been shown to be crucial for the endovascular invasion of trophoblast, as its deletion results in defective vascular remodelling and consequent embryonic death (Roca and Adams, 2007; Hunkapiller et al., 2011). Hence, these data demonstrate the important role of membrane ERα in maternal tissues for orchestrating expression of Notch family members and balancing angiogenic factors in order to support a correct trophoblastic expansion and development.

Moreover, we observed that the placental labyrinth, where the feto-maternal nutrient and gas exchanges occur, does not exhibit any morphological and size abnormalities in the placentas of C451A-ERα mothers at E14.5, as evidenced by histology and specific immunolabelling. Additionally, the placental oxygen saturation, assessed by photoacoustic imaging, was not reduced in mutant mothers, suggesting an altogether normal diffusional exchange between maternal and foetal circulations after embryo survival. However, these surviving embryos displayed increased placental size and weight at E14.5. In mice, placental weight inversely correlates with the number of *concepti* per litter (McLaren, 1965). Thus, the reduced number of implanted embryos might partially explain the hypertrophic placental phenotype. Nevertheless, histomorphological analyses also revealed a specific expansion of the junctional zone, associated with selective dilation of its vascular tree. The structural changes in Jz in the fully functioning mutant placentas of the surviving embryos probably result from the earlier reduced expansion of *Tpbpa*-positive trophoblasts, potentially affecting the normal placental vascularization and development, even though this may appear counter-intuitive. Consequently, the dilation of the vascular tree might appear to compensate for previously occurring suboptimal vascular conditions in order to increase the blood supply to the surviving embryos. It is also important to note that this Jz also constitutes the main endocrine compartment of the placenta that produces vast amounts of hormones and growth factors that are important for the normal progression of the pregnancy and can also explain the modified endocrine environment (Woods et al., 2018).

These regulations of trophoblast differentiation and invasiveness causing the spiral arterial remodelling by oestrogens have been evidenced in human and non-human primate physiological and pathological placentations (Bonagura et al., 2012; Berkane et al., 2017). In mice, this question received limited attention considering that all mouse models invalidated for oestrogen receptors are infertile. Therefore, C451A-ERα appears to be the first murine model to allow the investigation of the link between oestrogen signalling and trophoblast function during early *in vivo* placentogenesis. The reduced trophoblastic activity reported here obviously begs the question of the identity of the maternal cell types responsible for this phenomenon. Among the important regulators of these dynamic cellular and molecular changes, E2 has been shown to influence a phenotypically distinctive lymphocyte population of maternal uterine natural killer (uNK) cells that regulate vascular remodelling within the endometrium and decidua by producing a range of soluble products, including angiogenic cytokines (such as angiopoietin 2 or CCL2) (Moffett and Loke, 2006; Gibson et al., 2015). Moreover, uterine glands also secrete some other important stromal uterine factors, such as LIF [leukaemia inhibitory factor, a member of the interleukin-6 (IL-6) family]. LIF is highly induced in response to the nidatory surge in ovarian oestrogens at the beginning of pregnancy and is essential for embryo implantation (Stewart et al., 1992; Kelleher et al., 2019). Whether secretion of this factor is altered in C451A-ERα remains an unanswered question. All the E2-induced coordinated crosstalk between maternal uNK, uterine glands and vessels could be altered by the C451A-ERα mutation that leads to the observed phenotype and should be explored in future studies.

Finally, delayed parturition occurring in average 4 days later than in wild-type mice is another observed phenotype of C451A-ERα mice. This delayed parturition probably explained the neonatal deaths of pups of C451A-ERα mothers as pre-term ultrasound imaging and caesarean dissection of embryos from C451A-ERα mice confirmed intra-uterine viability of foetuses at term and the post-natal survival of pups delivered by caesarean sections. Although initial reports mentioned that a major signal for proper labour initiation in rodents is a higher E2/progesterone ratio at term, the observed imbalance in favour of progesterone in C451A-ERα mice likely explains the delayed parturition that results in litter loss (Kota et al., 2013; Ilicic et al., 2020). Indeed, C451A-ERα mothers display a failure in the expected pre-partum decline in progesterone concentration, as attested by a 16-fold increase in its levels compared with their wild-type littermates. Therefore, excessive progesterone in C451A-ERα mothers might prolong the relaxation of the myometrium and prevent the E2-mediated uterine contractility needed for labour induction. This observation is in agreement with the pre-term parturition induced in wild-type mice treated with the progesterone antagonist RU486, while progesterone treatment delayed parturition (Edey et al., 2018). Consequently, these data suggest that membrane ERα participates in the regulation of the endocrine mechanisms of labour induction in mice.

In conclusion, this study uncovers important pathways regulated by membrane ERα signalling during pregnancy. Evidence of the crucial effects of the membrane-initiated ERα signalling in the regulation of expansion of trophoblast cell populations, which is crucial for spiral arterial remodelling, are provided for the first time. In addition, our experiments document the crucial role of membrane-initiated ERα signalling in the regulation of endocrine mechanisms involved in mouse parturition. Further determination of the molecular mechanisms behind the actions of ERα may elucidate a unifying scheme of placental development and its regulation by oestrogens to open new avenues for the research of human pregnancy pathologies.

## MATERIALS AND METHODS

### Animals and general procedures

The C451A-ERα knock-in mouse line was generated on a C57Bl/6 background at the Mouse Clinical Institute as previously described (Adlanmerini et al., 2014). All experimental procedures were approved by the local Ethical Committee of Animal Care and conducted in accordance with the guidelines established by the Institut National de la Santé et de la Recherche Médicale (INSERM) and the Belgian law on the ‘Protection and welfare of experimental animals’. The experiments were carried out in the animal facilities of the University of Toulouse (Protocol 2019012118144570) and the University of Liège (Animalerie Centrale, agreement number LA1610002; Protocol 1620). All animals had free access to food and water. Animals were housed under a normal 12 h light/dark cycle (lights on at 07:00 am) for all experiments. All experiments were carried out on 2- to 4-month-old females.

To evaluate gestational success, females (C57Bl/6 WT-ERα, *n*=10; C451A-ERα, *n*=12) were placed overnight with a WT-ERα male for 5-7 consecutive days, and mated three times over a period of 4 months. Following each mating session, the number of females who gave birth, as identified by the presence of pups in their nest, and the number of live and dead pups per litter were counted. Following the second and third mating sessions, the presence of a vaginal plug was checked to confirm mating on the following morning [day post plug (E0.5)] and females with a plug were weighed daily between 08:00 and 10:00 am for 27 days. Weight monitoring was carried out in two additional cohorts of females (C57Bl/6 WT-ERα, *n*=32; C451A-ERα, *n*=26) to provide sufficient statistical power to the analysis. Some of these females were also monitored by ultrasound imaging at embryonic day E9.5, E14.5 and E18.5. Mice were considered to be pregnant if they had gained more than 1 g on E7.5, and as non-pregnant if they had gained less than 1 g on E7.5. Parturition was monitored after E16.5 twice a day. Parturition timing was defined as the observation of a first pup in the nest or weight loss. The viability of pups was also evaluated.

### Spontaneous ovulation

Females were mated overnight with a WT-ERα male once a week for 5 weeks and checked for a vaginal plug the next morning. Females (WT-ERα, *n*=18; C451A-ERα, *n*=14; C57Bl/6) with a plug were killed by cervical dislocation within 3 h of vaginal investigation. The infundibulum was opened with a needle and the number of ovulated oocytes was counted in each uterine horn.

### Transcutaneous ultrasound imaging during gestation

A multifrequency ultrasound biomicroscope (model Vevo 2100, Imaging platform and Vevo Imaging station, VisualSonics) was used to determine the presence of live and dead embryos or foetuses in the same mothers repeatedly imaged at E9.5, E14.5 and E18.5. MS550D and MS700 were used for E9.5 ultrasound and for both E14.5 and E18.5 ultrasound, respectively, as these devices are better adapted to image deep and superficial tissue, respectively. Transcutaneous imaging of embryos and foetuses was performed at 40 MHz. Briefly, on E8.5, the abdomen of the mother was shaved with a chemical hair remover to minimize ultrasound attenuation. After anaesthesia with 5% of isoflurane (IsoFlo, B506, Zoetis, Belgium) and maintained at 2.5% during application of prewarmed ultrasound gel (Rodisonic contact gel ultrasound transmission gel, Pannoc, Belgium), embryos (E9.5) and foetuses (E14.5 and E19.5) were examined, counted and considered as alive by the identification of a heartbeat.

### Photoacoustic imaging

Pregnant mice (*n*=5 per genotype) were anaesthetised by 2% isoflurane inhalation. Body temperature and respiration rate were monitored and maintained throughout the experiment. Three-dimensional photoacoustic Imaging (PAI) (Vevo LAZR-X, Visualsonics) was used to obtain whole placenta and foetus oxygen saturation (SO2) mapping. Based on the conversion of pulsed laser at 750 and 850 nm excitation wavelengths into ultrasonic emission, the relative concentration of oxygenated and deoxygenated haemoglobin (HbO2 and Hb, respectively) was calculated. Images were obtained using a 20 MHz central frequency probe (MX250). A medium optical fibre transmitting a tunable laser with a peak pulse energy output of 50-60 mJ/cm^2^ was used (David et al., 2020). The oxy-haemo mode was associated with PA gain at 34 dB and imaging depth at 24 mm for every acquisition. The fusion of anatomical images, obtained using high-resolution ultrasound (B-mode) with PAI data were analysed offline using the Vevo LAB software (v5.5.1). Analyses were performed on two to five live placentas from each mother at E14.5, as described previously (Lawrence et al., 2019).

### Histology

For tissue collection at E14.5, females mated with a WT-ERα male that had gained more than 1.5 g by E10 of the pregnancy were euthanized at E14.5 by cervical dislocation. In each uterine horn, the number of viable and aborted foetuses was counted. Resorptions were identified as round-shaped haemorrhagic masses of small size (0.02-0.1 g), where embryonic development had stopped prematurely (Fig. S2). Crown-rump distance and the presence of external anomalies were also evaluated to assess the growth and development of foetuses. In addition, placental diameters and weights were measured.

*Concepti* at E9.5 or collected placentas at E14.5 were fixed for 2 h in 4% PFA, cross-sectioned in the middle and embedded in paraffin wax. Sections (5 µm) were collected at 15 μm intervals and stained with Haematoxylin and Eosin. Images were acquired using a NanoZoomer Digital Pathology Scanner. NDP View software (Hamamatsu 470 Photonics) was used for quantification. The area of each placental layer (decidua basalis, junctional zone and labyrinth) was measured on the central sections of 38 placentas obtained from 16 mothers (WT-ERα, *n*=6; C451A-ERα, *n*=10).

### Immunofluorescence

Fresh frozen (12 µm) or paraffin wax-embedded (5 µm) serial placental sections were immunostained using anti-CD31 (BD550274, BD Pharmingen, 1:50), anti-MCT1 (Ab1286-I, Millipore Sigma, 1/1000), anti-MCT4 (Ab3314-P, Abcam, 1:400), anti-Tpbpa (Ab104401, Abcam, 1:200) and anti-Ck8 (Troma-I, Developmental Studies Hybridoma Bank, 1:7) antibodies. Images were acquired using a NanoZoomer Digital Pathology Scanner and NDPView software (Hamamatsu Photonics). The precise middle sections of E9.5 whole implantations were identified in the serial sections and the Tpbpa-positive cell surface was quantified using NDPView 2 software.

### Measurement of the arterial diameter of the uterine artery

At E9.5, animals were sacrificed by CO_2_ inhalation. The uterine artery was carefully collected and dissected in ice-cold physiological salt solution (PSS) of the following composition (mmol/l): 130.0, NaCl; 15.0, NaHCO_3_; 3.7, KCl; 1.6, CaCl_2_; 1.2, MgSO_4_; and 11.0, glucose. Two arterial segments (cervix and ovary side) were cannulated between two glass pipettes and bathed in PSS (pH 7.4) (PO_2_ 160 mmHg and PCO_2_ 37 mmHg). The pressure was controlled by a servo-perfusion system and diameter changes were measured continuously. Arterial viability was tested as follows: (1) arterial contractility was tested with phenylephrine (1 nM to 10 µM) and (2) endothelial-dependent relaxation was tested with acetylcholine (1 pM to 10 µM) after phenylephrine-induced (1 µM) precontraction. The PSS was then changed for a Ca^2+^-free PSS containing ethylenbis-(oxyethylenenitrolo) tetra-acetic acid EGTA (2 mM), papaverin (100 µmol/l) and sodium nitroprusside (10 µM). The arterial diameter was determined in response to stepwise increases in intraluminal pressure from 10 to 125 mmHg using a video-monitored perfusion system (LSI) and the passive diameter was recorded for each pressure step (Kauffenstein et al., 2016).

### Quantitative real-time PCR

Total RNA was extracted from fresh frozen implantation sites using TRIzol reagent (Ambion Life Technologies). Samples were obtained at E9.5, from seven WT-ERα and six C451A-ERα mothers mated with WT-ERα mice. 1 μg of total RNA was used to synthesize cDNA (Applied Biosystems-Thermo Fisher Scientific). Assays were performed in duplicate, using a Real-Time PCR system (SsoFast EvaGreen Supermix). Quantitative RT-PCR analysis of different genes was performed. All the primers for real-time PCR are listed in Table S2.

### Hormonal level measurement

Blood samples were collected from the left ventricle of anaesthetised pregnant mice (E18.5), via puncture on the left side of the chest from the top of the sternum. Samples were collected in clotting activating microtubes (1.1 ml Z-Gel-Sarstedt), centrifuged (5 min at 1000 rpm) and kept at −80°C until they underwent hormone assays. Circulating levels of oestradiol (E2) and progesterone were measured simultaneously by gas chromatography coupled with mass spectrometry (GC-MS) on 200 µl of serum, as previously described (Giton et al., 2015).

### Statistics

Data were analysed with Prism 8 (GraphPad) and are reported in Table S3. Continuous data were analysed using a parametric unpaired Student's *t*-test, two-way ANOVAs or a non-parametric Mann–Whitney test when normality and/or homoscedasticity assumptions were violated. Significant ANOVA results were followed by Tukey's post-hoc tests. qPCR data were analysed by nested *t*-tests and are represented by violin plots, each representing data obtained from three to five placentas from the same mother. Contingency data were analysed by Fisher's exact tests. Results were considered significant when *P*<0.05 (*P<0.05, **P<0.01 and ***P<0.001). All results are represented as mean±s.e.m. unless mentioned otherwise.

## Supplementary Material

10.1242/develop.200683_sup1 Supplementary information
