# Supplementary material for: Loss of function of the maternal membrane oestrogen receptor ERα alters expansion of trophoblast cells and impacts mouse fertility
Source: Development. 2022 Oct 13;149(19):dev200683. doi: 10.1242/dev.200683 (PMC9720743; doi:10.1242/dev.200683)
Supplement: Supplementary information [file develop-149-200683-s1.pdf]

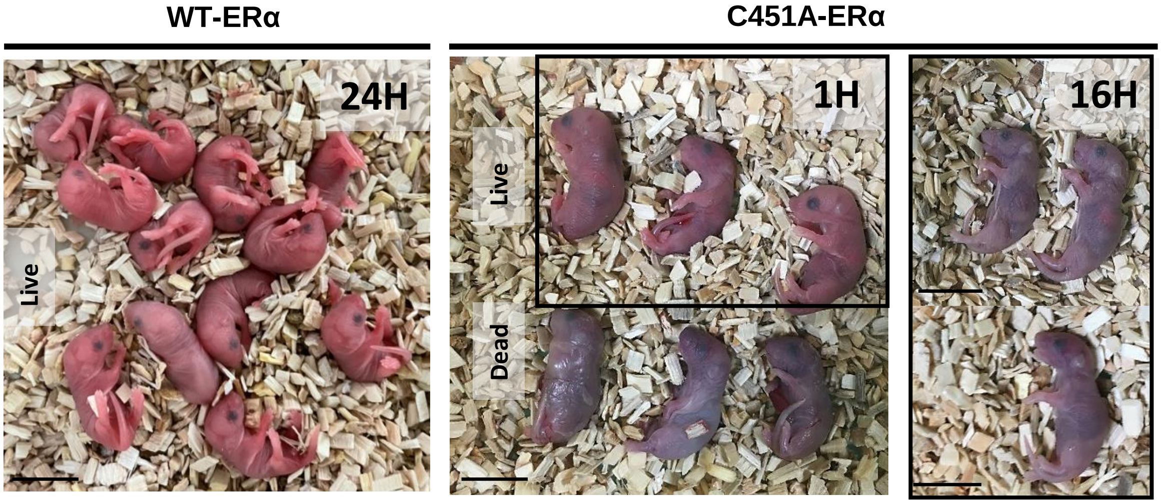

Fig. S1. Phenotype of newborns from WT-ERα and C451A-ERα mice.
Data demonstrate complete neonatal mortality of pups born in nests from C451A-ERα mothers within a few hours from birth on postnatal day 1, while no apparent congenital abnormalities are observed.

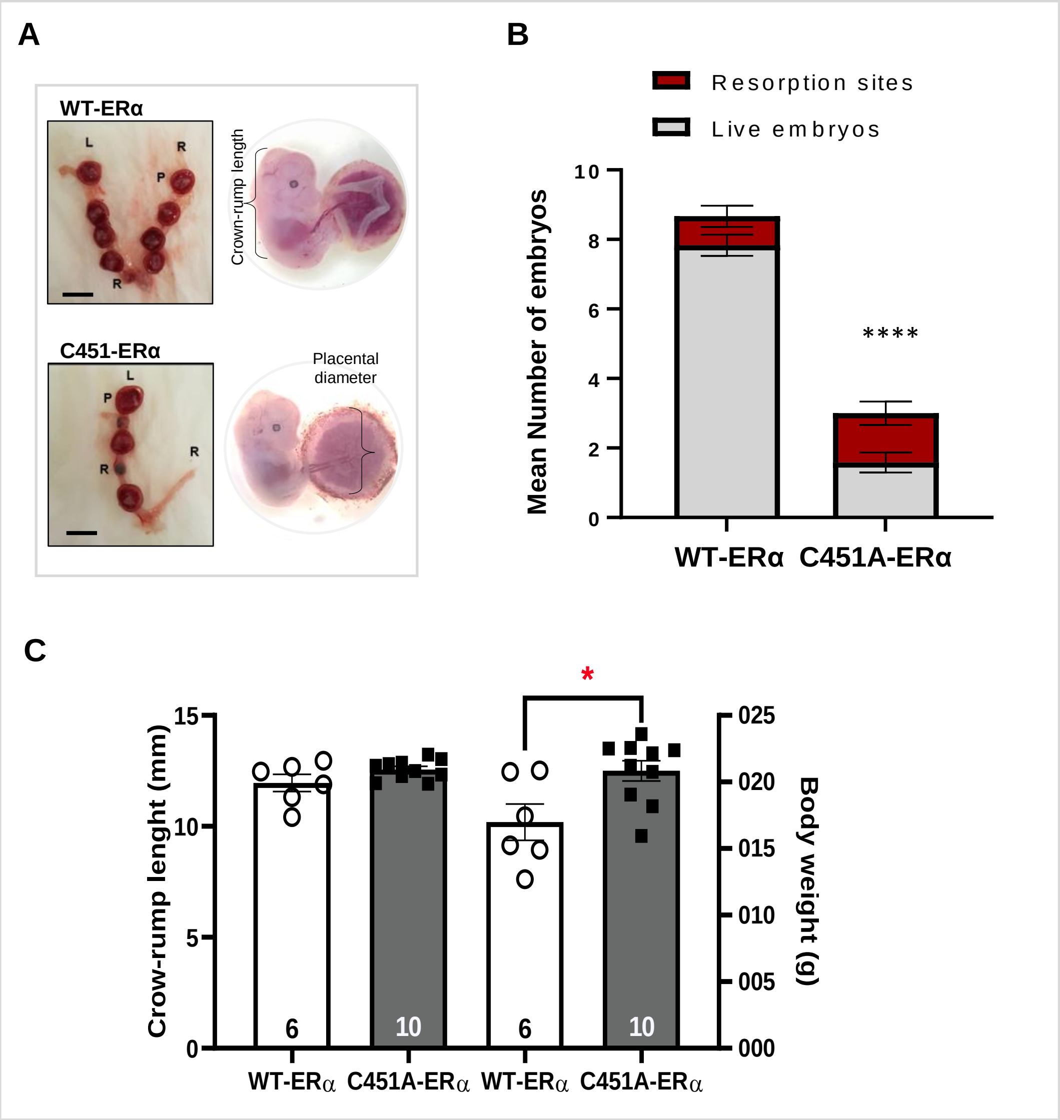

Fig. S2. Representative macroscopic images of reconstructed pregnant uteri of WT-ERα and C451A-ERα mice at E14.5 after fetus dissections, showing placentas (P) and resorption sites (R), corresponding to previously aborted embryos. Fetuses dissected and removed from the parietal yolk are represented separately. B) Quantification of total numbers of implanted embryos collected in uteri of C451A-ERα (n= 6) and WT (n=12) mice, with respective numbers of live and aborted embryos. **** P<0.001, 2-way ANOVA. C) Crow-rump length and Body weight of embryos at E14.5; Mann Whitney test, * P<0.05

Development • Supplementary information

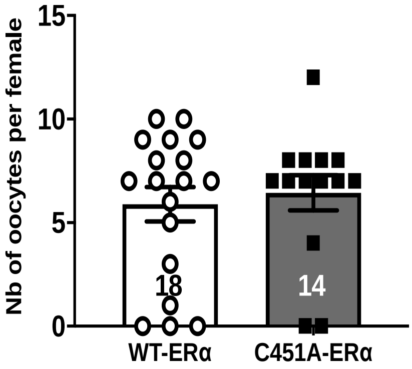

Fig. S3. No difference in ovulation rate was observed between WT-ERα and ERα-C451A mice following mating

Number of oocytes collected in naturally cycling female after being mated overnight with a WT-ERα male (WT-ERα, n=18; C451A-ERα, n=14). Unpaired student t test (ns).

Development • Supplementary information

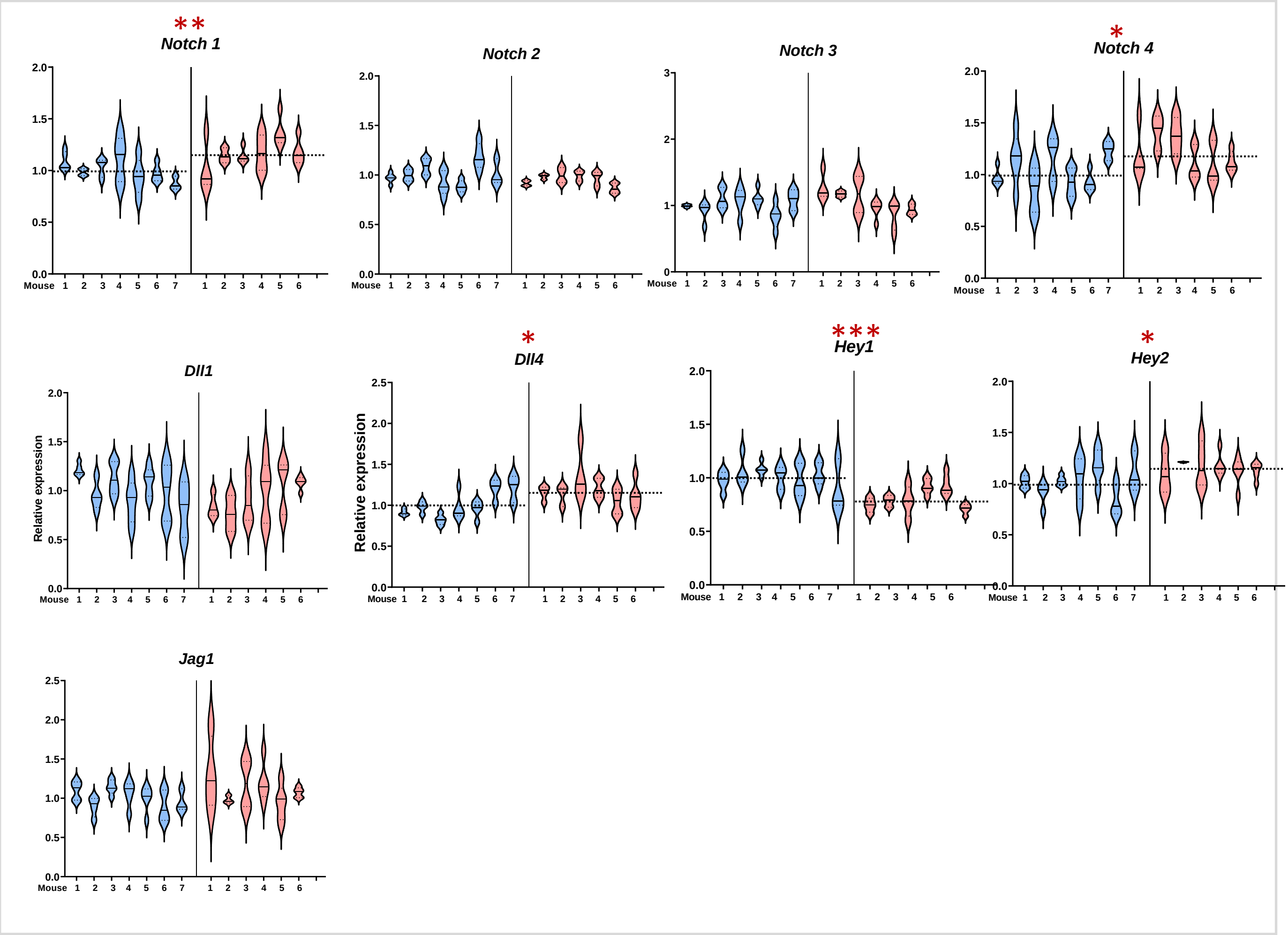

Fig. S4. Gene expression profile in implantation sites of C451A-ERα and WT-ERα mothers at E9.5.

Real time RT-qPCR for, *Pcdh12, Cts7, Cts8, Mmp2, Mmp9, Timp1, Notch2, Notch3, Dll1, Jag1, Pecam1 and Vcam1* genes in implantation sites (E9.5) of WT-ERα and C451A-ERα mouse. Expression level of listed genes does not differ between the genotypes. Data included 59 placental samples, obtained from 7 WT-ERα and 6 C451A-ERα mice. Each violin plot represents a distribution of data corresponding to 3-5 placental samples obtained from the same mother. *P<0.05 Nested t test.

Table S1. Circulating hormonal levels in pregnant WT-ERα and C451A-ERα mice at E18.5

E18.5	E2	Testosterone	D4-Dione	Progesterone	Androsterone	DHT	DHEA
**WT-ERα**	24,85±3,9	0,33 ± 0,09	0,17 ± 0,03	8,85 ± 5,33	0,58 ± 0,10	0,09 ± 0,02	0,15 ± 0,03
**C451A-ERα**	36,4±7,6	0,44 ± 0,10	1,09 ± 0,30	134,34 ± 28,7***	1,31 ± 0,21*	0,30 ± 0,15	0,19 ± 0,04*
				*******	*****		*****
				p=0,00015	***p=0,0207***		***p=0,0104***

Circulating levels of 17β-Estradiol **(E2)**, Estrone **(E1)**, Progesterone, Dihydrotestosterone **(DHT)**, Androsterone, Testosterone, Androstenedione **(D4-Dione)**, dehydroepiandrosterone **(DHEA)** in pregnant WT-ERα and C451A-ERα mice at embryonic 18.5 (E18.5).

At E18.5 C451A-ERα mice exhibit higher circulating levels of Progesterone, Androsterone and D4-Dione, compared to control females. *p<0.05; ***p<0.001 Mann Whitney test compared to WT-ERα on the same embryonic day. WT-ERα (n=8) and C451A-ERα mice (n=8). Results are presented as mean ± SEM.

Development • Supplementary information

Table S2. Primers used for RT-qPCR

Target	Forward	Reverse
Tpbpa	CCAGCACAGCTTTGGACATCA	TCTTCATAAACTTACTCCACACTGC
Prl4a1	ACCACCAGATGCCACACTTCT	GTAGACTGTGTGCCAACCCCA
Prl7b1	GCCTTTTCCAGTGCCCCACAA	ACGTCCTCCCACAGGAGAAGAT
Prl3d	GTGCCCACTGAAGACCTGTAT	GACACAGGGGAAGTGTTCTGTCT
Prlc2c	GAACAAGCCAGGCTCACACA	CCAGGCATCCAAAATCATGGC
Pcdh12	AAGAGCTGTCGAGCCTGTTGG	ACGTTGTAGCATCGGGGGAAG
Esr1	CTGTCGGCTGCGCAAGTGTT	AACATTCTCCCTCCTCGGCGGT
Pgr	GCGGTGCTCAAGGACAGCCT	ACTGTGGGCTCTGGCTGGCT
Vegfa	CAGCAGATGTGAATGCAGACCAA	CTTTCTCCGCTCTGAACAAGGC
FLT	TCACCAAGCTTTGATGGCATTC	GCATTAAATCTCCACTCACAATAGCC
Pgf	ACGACAAAGGCAGAAAGGAGGA	GGCTGGTTACCTCCGGGAAAT
Egfl7	AGAGGAGGTGTACAGGCTGCA	TTCGGTCCAGCTGCTGGAAGGAAT
Vegfr2	TCCGGAAGCTCTGATGATGTGA	AGTGTCCAGCTGATAGTCCTCA
sFlt1	TCACCAAGCTTTGATGGCATTC	GCATTAAATCTCCACTCACAATAGCC
Egfr	CACTGTGTCAAGACCTGCCCA	ACTGCCATTGAACGTACCCAGA
Notch1	AATGGAGGGAGGTGCGAAGTG	TTGGAGTCCTGGCATCGTTGG
Notch2	TGCAGTGTCGAGGTGGTCAAG	TCCCAAGAAGCCCTCTGGACA
Notch3	TCCTCCTCAGGGAGATGCAGA	AAGTGGGGTGAAGCCATCAGG
Notch4	CCTGGACCTGCTTGCAACCTT	TCAGGCAGGGATTAGAGGTGC
Dll4	TTGCCCTTCAATTTCACCTGGC	TCTTACCCACAGCAAGAGAGCC
Hey1	TGTTCCATGTCCCCAACGACA	TCTCGATGATGCCTCTCCGTCT
Hey2	TGAAGCGCCCTTGTGAGGAA	ATCACTGAGCTTGTAGCGTGC
Ang 2	GCACCGCTAACCAACCAAAG	ACACGGCCTTGTCATTGTGA

Development • Supplementary information

Table S3. Results of statistics

Figure	n	Test		p	post-hoc
1-A<br>Weight gain	WT-pregnant (25)<br>WI-pregnant (16)<br>KI aborted (8)<br>non pregnant (28)	2-way<br>ANOVA	Group, $F_{3,73}$ = 63.41<br>Day, $F_{14,1022}$ = 274.4<br>Interaction, $F_{14,1022}$ = 81.87	p<0.001<br>p<0.001<br>p<0.001	Tukey
1-B<br>Weight gain	WT-pregnant (16)<br>WI-pregnant (11)<br>non pregnant (22)	2-way<br>ANOVA	Group, $F_{2,46}$ = 89.19<br>Day, $F_{13,598}$ = 46.74<br>Interaction, $F_{26,598}$ = 33.66	p<0.001<br>p<0.001<br>p<0.001	Tukey
1-C<br>Length of gestation	WT (17)<br>KI (11)	Mann-whitney	U=3.5	p<0.0001	
1-D (left)<br>Number of pups	WT (7-8)<br>KI (2-5)	Mann-whitney	T1, U=0.5, n_1=7, n_2=4<br>T2, U=2; n_1=8, n_2=5<br>T3, U=2.5; n_1=7, n_2=2	p=0.0091<br>p=0.0031<br>p=0.2778	
1-D (middle)<br>Number of viable pups	WT (7-8)<br>KI (2-5)	Mann-whitney	T1, U=0; n_1=7, n_2=4<br>T2, U=0; n_1=8, n_2=5<br>T3, U=0; n_1=7, n_2=2	p=0.0061<br>p=0.0008<br>p=0.0566	
1-D<br>(right)<br>% Pregnant females	WT (7-8)<br>KI (2-5)	Fisher's exact	T1<br>T2<br>T3	p=0.1984<br>p=0.0991<br>p=0.0092	
1E: placental weight at E18.5	WT (8)<br>KI (8)	Mann-Whitney	U=7	p= 0.0067	
1F: Offspring weight at E18.5	WT (8)<br>KI (8)	Mann-Whitney	U=20	p=0.2345	
1G (left):<br>E2 serum levels-<br>E18.5	WT (8)<br>KI (8)	Mann-Whitney:	U=22	P=0.3282	
1G (right):<br>progesterone serum levels	WT (8)<br>KI (8)	Mann-Whitney:	U=0	p=0.0002	
2-A:<br>Ultrasound<br>Total embryos at E9.5	WT (17)<br>KI (11)	Mann-whitney:<br>total	U=44	p=0.0172	
2-B:<br>Ultrasound<br># embryo/mother E14.5	WT (17)<br>KI (12)	Mann-whitney:<br>total<br>Live	U=64<br>U=19	p=0.0484<br>p<0.0001	
2-C:<br>Ultrasound# fetuses<br>E18.5	WT (17)<br>KI (13)	Mann-whitney:<br>total<br>Live	U=34<br>U=19	p=0.0016<br>p<0.001	
3-A % of Resorptions E14.5	WT (6)<br>KI (12)	Mann-Whitney	U=15	p=0.0467 *	
3-B:<br>Postmortem Placenta size and weight E14.5	WT (6)<br>KI (10)	Mann-Whitney:<br>Thickness<br>Diameter<br>Weight	U=25<br>U=8<br>U=0.5	p=0.6354<br>p=0.0004<br>p=0.016	

Development • Supplementary information

3C: JZ area E14.5	WT-ERα (6)<br>C451A-ERα (10)	Mann-Whitney:	U=9	p=0.0225	
3D: Lab Area E14.5	WT-ERα (6)<br>C451A-ERα (10)	Mann-Whitney:	U=17	p=0.1806	
3G: Vascular channel E14.5	WT-ERα (6)<br>C451A-ERα (10)	Mann-Whitney	U=2	p=0.001	
3I: SO2 placenta at E14.5	WT-ERα(5)<br>C451A-ERα (5)	Mann-Whitney	U=6	p=0.2222	
4-B-C Uterine arterial remodeling	Uterine artery<br>WT-ERα Virgin (5)<br>C451A-ERα virgin (8)<br>WT-ERα pregnant (4)<br>C451A-ERα pregnant (3)<br><br>Ovarian artery<br>WT-ERα Virgin (4)<br>C451A-ERα virgin (5)<br>WT-ERα pregnant (4)<br>C451A-ERα pregnant (3)	Repeated measure 2 way ANOVA	Pressure: $F_{5,80}$=330.5<br>Group: $F_{3,16}$=12.18<br>Interaction: $F_{15,80}$=6.307<br><br>Pressure: $F_{5,75}$=341.2<br>Group: $F_{3,15}$=13.76<br>Interaction: $F_{15,75}$=6.070	p<0.001<br>p=0.002<br>p<0.001<br><br>p<0.001<br>p=0.001<br>p<0.001	Tukey
5- Gene expression by RT-qPCR E9.5	WT (7)<br>KI (6)<br>3 to 4 embryos per female (59 samples)	Nested t test:<br>Tpbpa<br>Prl7b1<br>Prl4a1<br>Prlc2c<br>Prl3d1<br>Pcdh12<br>Esr1<br>Pgr<br>PlGF<br>sFlt1<br>VEGFa<br>VEGFR1<br>VEGFR2<br>Ang2<br>EGl7	Tpbpa t_{11}=3.439<br>Prl7b1 t_{11}=3.202<br>Prl4a1 t_{11}=2.258<br>Prlc2c t_{11}=2.642<br>Prl3d1 t_{11}=2.702<br>Pcdh12 t_{11}=0.5249<br>Esr1 t_{11}=1.296<br>Pgr t_{11}=0.3488<br>PlGF t_{11}=1.323<br>sFlt1t_{11}=2.359<br>VEGFa t_{11}=1.070<br>VEGFR1 t_{11}=2.0169<br>VEGFR2 t_{11}=0.6869<br>Ang2 t_{11}=4.589<br>EGl7 t_{11}=3.684	Tpbpa p=0.0055<br>Prl7b1 p=0.0084<br>Prl4a1 p=0.0452<br>Prlc2c p=0.0229<br>Prl3d1 p=0.0206<br>Pcdh12 p=0.6101<br>Esr1 p=0.2215<br>Pgr (P=0.7338 ns)<br>PlGF p=0.2125<br>sFlt1 p=0.0379<br>VEGFa p=0.3077<br>VEGFR1 p=0.052	

				VEGFR2 p=0.5064<br>Ang2 p=0.0008<br>EGl7 p=0.036	
6-B Placental surface E9.5	WT-ERα (5)<br>C451A-ERα (5)	Mann-whitney	U=1	p=0.0159	
6-C Thickness uteri-placental unit E9.5	WT-ERα (5)<br>C451A-ERα (5)	Mann-whitney	U=1	p=0.0159	
6-E Tpbpa positive cell surface E9.5	WT-ERα (6)<br>C451A-ERα (5)	Mann-whitney	U=3	p=0,0260	
S2-B Nb live & aborted embryos E14.5 (post-mortem)	WT(6)<br>KI (12)	Mann Whitney<br>Resorptions<br>Live embryos	U=0<br>U=0	p<0.001<br>p<0.001	
S2-C Post-mortem embryo size E14.5	WT (6)<br>KI (10)	Mann Whitney	Crown-rump length U=17<br>Fetuses body weight U=9	p=0.1806<br>p=0.0225	
S3 Nb oocytes	WT (14)<br>KI (18)	Student t test	t_{30}=0.4465	p=0.6585	
S4-RT-qPCR-E9.5	WT (7)<br>KI (6)<br>3 to 4 embryos per female (59 samples)	Nested test:<br>Notch1<br>Notch2<br>Notch3<br>Notch4<br>Dll1<br>Dll4<br>Hey1<br>Hey2<br>Jag1	Notch1 t_{11}=3.107<br>Notch2 t_{11}= 1.16<br>Notch3 t_{11}= 0.46<br>Notch4 t_{11}= 2.432<br>Dll1 t_{11}= 1.014<br>Dll4 t_{11}= 2.324<br>Hey1 t_{11}= 4.451<br>Hey2 t_{11}= 2.618<br>Jag1 t_{11}= 1.554	Notch1 P=0.01<br>Notch2 P= 0.2705<br>Notch3 P=0.6545<br>Notch4 P=0.0333<br>Dll1 P=0.3322<br>Dll4 P=0.403<br>Hey1 P=0.001<br>Hey2 P=0.0239<br>Jag1 P= 0.1484	

Click here to download Table S3

Development • Supplementary information
