# Supplementary material for: Loss of function of the maternal membrane oestrogen receptor ERα alters expansion of trophoblast cells and impacts mouse fertility
Source: Development. 2022 Oct 13;149(19):dev200683. doi: 10.1242/dev.200683 (PMC9720743; doi:10.1242/dev.200683)
Supplement: Supplementary file 1 [file develop-149-200683_review_history.pdf]

Loss of function of maternal membrane oestrogen receptor ERα alters expansion of trophoblast cells and impacts mouse fertility

Mariam Rusidzé, Mélanie C. Faure, Pierre Sicard, Isabelle Raymond-Letron, Frank Giton, Emilie Vessieres, Vincent Prevot, Daniel Henrion, Jean-François Arnal, Charlotte A. Cornil and Françoise Lenfant
DOI: 10.1242/dev.200683

Editor: Liz Robertson

Review timeline

Original submission:	1 March 2022
Editorial decision:	18 May 2022
First revision received:	22 August 2022
Accepted:	31 August 2022

Original submission

First decision letter

MS ID#: DEVELOP/2022/200683

MS TITLE: Loss of function of Membrane Estrogen Receptor ERÎ± alters expansion of trophoblast cells and impacts mouse fertility

AUTHORS: Mariam Rusidze, Melanie Faure, Pierre Sicard, Isabelle Raymond-Letron, Frank GITON, Emilie Vessieres, Vincent PREVOT, Daniel HENRION, Jean-Francois ARNAL, Charlotte Cornil, and Francoise LENFANT

Please accept my sincere apologies for the unacceptably long delay in having your paper reviewed at Development. Unfortunately I had a lot of difficulty finding reviewers and then the situation was further exacerbated as one of the reviewers has failed to summit their comments inspite of repeated requests and reminders from the Office staff and myself. I have decided to proceed based on the single, thorough, review I have received, since the comments very much overlap with those I would have raised. The referee comments are appended below, or you can access them online: please go to BenchPress and click on the 'Manuscripts with Decisions' queue in the Author Area.

As you will see, they express considerable interest in your work, but have some significant criticisms and recommend a substantial revision of your manuscript before we can consider publication. If you are able to revise the manuscript along the lines suggested, which may involve further experiments, I will be happy receive a revised version of the manuscript. Your revised paper will be re-reviewed by the original referee, and acceptance of your manuscript will depend on your addressing satisfactorily the major concerns they raise. Please also note that Development will normally permit only one round of major revision. If it would be helpful, you are welcome to contact us to discuss your revision in greater detail. Please send us a point-by-point response indicating your plans for addressing the referee’s comments, and we will look over this and provide further guidance.

Please attend to all of the reviewers' comments and ensure that you clearly highlight all changes made in the revised manuscript. Please avoid using 'Tracked changes' in Word files as these are lost in PDF conversion. I should be grateful if you would also provide a point-by-point response detailing

© 2022. Published by The Company of Biologists under the terms of the Creative Commons Attribution License (https://creativecommons.org/licenses/by/4.0/).

how you have dealt with the points raised by the reviewers in the 'Response to Reviewers' box. If you do not agree with any of their criticisms or suggestions please explain clearly why this is so.

Reviewer 1

Advance summary and potential significance to field

This article describes the effect of knocking out the maternal membrane-localized isoform of the estrogen receptor α on pregnancy, placental development and parturition in mice. I found it a fascinating read, and I suspect other readers will too. It's an intriguing phenotype, and reveals some interesting aspects of uterine-placental communication. The phenotyping is highly detailed examining multiple stages and multiple aspects of placental development and spiral artery remodeling in detail. The methods are appropriate, and the approach is logical. Particularly interesting is the demonstration that membrane receptor estrogen signaling in maternal cells is necessary for placental cell proliferation/differentiation and uterine artery remodeling. The observation of what looks like impaired vascular drainage from the junctional zone is not something I've seen before.

Comments for the author

I do have some major concerns about the presentation and interpretation of data, which are easily remedied.

- First and foremost, placental trophoblast and endothelium are embryo-derived, and, because the sires used here were wildtype, the placental cells all have at least one normal ESR1 copy. Thus, the experiments described test the effect of membrane ESR1 IN MATERNAL CELLS (probably uterus?) on placental development, not membrane ESR1 action within placental cells. This needs to be stated in the abstract, the introduction and carefully worded throughout. The phrases "C451A-Erα placentas" and "mutant mouse placentas, compared to their WT littermates" are particularly misleading, whereas wording like "placentas from mothers of both genotypes" is quite clear. Another example is on line 308 "Thus, the membrane function of ERα [in maternal cells] is required for the SpA-TGCs expansion through the maternal vascular unit and therefore for the control of spiral arterial remodelling". Several statements in the discussion also elide the distinction between estrogen signaling in the placenta and signaling in the uterus, with downstream effects on placental cells.
- Similarly, because the Tpbpa -lineage trophoblast are not mutant, the authors should speculate in the discussion on which maternal cell types could be responsible for the loss of these trophoblast and their invasion (uNKC? Spiral artery endothelium? Glandular epithelium?). I actually think this is one of the most interesting questions raised by the results.
- For readers not familiar, the introduction should describe the C451A-ERa mouse model in a bit more detail, e.g that palmitoylation at C451 is required to traffic ERa to the membrane, and summarize the effect of C451A on membrane and nuclear ERa signaling/activation. It needs to acknowledge the differences between the two extant models with C-to-A substitution at C451. For this one, the C451A-ERa , this group previously reported ~60% reduction in membrane ERa whereas the Pedram/NOER paper described complete loss of membrane ERa. The NOER mice are acyclic and anovulatory, with thinner uterine lining, while C451A-ERa has at least normal numbers of (still abnormal) primary-tertiary follicles, a very few CL, and uteri that seem to respond normally by proliferating in response to E2 treatment. It's important to acknowledge that the C451A-ERa is probably not a complete knockout.
- In the introduction, the essential role of estrogen in embryo implantation via stimulation of LIF release from uterine glands should be acknowledged. Likewise, the discussion should consider whether this role of estrogen is at all responsible for the phenotype of failed implantation sites at 9.5.
- The observation of increased placental weight/area without increased fetal weight, and an increase in the diameter and appearance of vascular channels in the junctional zone suggests a problem with venous drainage from the placenta, causing edema. A few major canals close to the center of the placenta carry maternal blood from the uterine spiral arteries through the junctional zone to the labyrinth, while a larger number of smaller spaces carry blood back from the maternal blood sinuses in the labyrinth to the maternal veins, and these are what appear to be dilated in the placentas from mutant dams. Again, this is kind of fascinating.

© 2022. Published by The Company of Biologists under the terms of the Creative Commons Attribution License (https://creativecommons.org/licenses/by/4.0/).

Additional minor comments:
Line 182 “certainly due to a progesterone withdrawal failure.” It would be more accurate to say it is likely due to progesterone withdrawal failure. Although the data clearly demonstrate a failure of progesterone withdrawal, they don’t actually prove that it is solely responsible for the delayed parturition. An experiment showing that progesterone inhibition prevents the delay would be needed for certainty.
On what basis are mice in figure 1 separated into pregnant/ not-pregnant/ aborted? If both “pregnant” and “aborted” were plug positive, but one gave birth to pups and one didn’t, then these don’t seem like quite the correct terminology (i.e. aborted is an assumption). If weight is being used to assign categories, then presenting weight data is circular reasoning…
Please use official nomenclature. In particular, ERα is now ESR1.
Line 93 I don’t think “invalidated” (i.e. disproved) conveys the intended meaning here. Maybe “inactivated”?
Typo on line 221: Sentence ends with “while”

First revision

<u>Author response to reviewers' comments</u>

Reviewer 1 Advance summary and potential significance to field This article describes the effect of knocking out the maternal membrane-localized isoform of the estrogen receptor α on pregnancy, placental development and parturition in mice. I found it a fascinating read, and I suspect other readers will too. It’s an intriguing phenotype, and reveals some interesting aspects of uterine-placental communication. The phenotyping is highly detailed, examining multiple stages and multiple aspects of placental development and spiral artery remodeling in detail. The methods are appropriate, and the approach is logical. Particularly interesting is the demonstration that membrane receptor estrogen signaling in maternal cells is necessary for placental cell proliferation/differentiation and uterine artery remodeling. The observation of what looks like impaired vascular drainage from the junctional zone is not something I've seen before.
We first want to thank you for your positive assessment of our work and your thorough reading of this manuscript.

Reviewer 1 Comments for the author I do have some major concerns about the presentation and interpretation of data, which are easily remedied.
•First and foremost, placental trophoblast and endothelium are embryo-derived, and, because the sires used here were wildtype, the placental cells all have at least one normal ESR1 copy. Thus, the experiments described test the effect of membrane ESR1 IN MATERNAL CELLS (probably uterus?) on placental development, not membrane ESR1 action within placental cells. This needs to be stated in the abstract, the introduction and carefully worded throughout. The phrases “C451A-Erα placentas” and “mutant mouse placentas, compared to their WT littermates” are particularly misleading, whereas wording like “placentas from mothers of both genotypes” is quite clear. Another example is on line 308 ”Thus, the membrane function of ERα [in maternal cells] is required for the SpA-TGCs expansion through the maternal vascular unit and therefore for the control of spiral arterial remodelling”. Several statements in the discussion also elide the distinction between estrogen signaling in the placenta and signaling in the uterus, with downstream effects on placental cells.
We thank the reviewer for raising this point that will help clarify these data and avoid potential misunderstanding. We have tried to clarify this point throughout the manuscript:
In the title page: Membrane ERα in placental development has been changed to “mERα in maternal tissues for pregnancy
- In the abstract, this point was mentioned modifying the sentence ” Hence, maternal loss of membrane ERα function clearly alters the activity of invasive trophoblast cells during placentogenesis.” by “Hence, loss of membrane ERα within maternal tissues clearly alters the activity of invasive trophoblast cells during placentogenesis.”

- in the statement: “ This study reveals how a point mutation of Estrogen Receptor ERα alters pregnancy revealing the crucial role of this receptor for placental development and delivery.” was

© 2022. Published by The Company of Biologists under the terms of the Creative Commons Attribution License (https://creativecommons.org/licenses/by/4.0/).

changed into " This study reveals how a point mutation of Estrogen Receptor ERα alters pregnancy revealing the crucial role of this receptor within maternal tissues for both placental development and delivery".
In the discussion, we also clearly mentioned on lines 355-356: "Therefore, this embryonic lethality must result from an alteration of the maternal signals (probably from uterus) for placental development in the ERα-C451A mice.
And on line 388 (previous line 308), the sentence has also been modified: "Altogether, these data demonstrate that the membrane function of ERα in maternal tissues is required for the SpA-TGCs expansion through the maternal vascular unit, hence for the control of spiral arterial remodelling."

We have carefully searched the manuscript for additional sources of potential confusion and have reworded accordingly.

•Similarly, because the Tpbpa -lineage trophoblast are not mutant, the authors should speculate in the discussion on which maternal cell types could be responsible for the loss of these trophoblast and their invasion (uNKC? Spiral artery endothelium? Glandular epithelium?). I actually think this is one of the most interesting questions raised by the results.
Indeed, this is a very interesting question that will require a lot of additional work. We therefore added a paragraph on lanes 459-471 to discuss this point. "Among the important regulators of these dynamic cellular and molecular changes, E2 was shown to influence a phenotypically distinctive lymphocyte population of maternal uterine natural killer (uNK) cells that regulate vascular remodeling within the endometrium and decidua by producing a range of soluble products, including angiogenic cytokines (such as angiopoietin-2 or CCL2) (Moffett and Loke, 2006; Gibson et al., 2015). Moreover, uterine glands also secrete some other important stromal uterine factors such as LIF (leukemia inhibitory factor, a member of the interleukin-6 (IL-6) family). LIF is highly induced in response to the nidatory surge in ovarian estrogens at the beginning of pregnancy and is known to be essential for embryo implantation (Stewart et al., 1992; Kelleher et al., 2019). Whether secretion of this factor is altered in C451A-ERα remains an open question. So, it is highly possible that all these E2-induced coordinated cross-talks between maternal uNK, uterine glands and vessels are altered by the C451A-ERα mutation, leading to the observed phenotype".

•For readers not familiar, the introduction should describe the C451A-ERa mouse model in a bit more detail, e.g that palmitoylation at C451 is required to traffic ERa to the membrane, and ummarize the effect of C451A on membrane and nuclear ERa signaling/activation. It eeds to acknowledge the differences between the two extant models with C-to-A ubstitution at C451. For this one, the C451A-ERa , this group previously reported 60% reduction in membrane ERa, whereas the Pedram/NOER paper described omplete loss of membrane ERa. The NOER mice are acyclic and anovulatory, with hinner uterine lining, while C451A-ERa has at least normal numbers of (still bnormal) primary-tertiary follicles, a very few CL, and uteri that seem to respond ormally by proliferating in response to E2 treatment. It's important to acknowledge hat the C451A-ERa is probably not a complete knockout.

Both models with membrane loss-of -function have been generated the same way, by mutating the palmitoylation site into an alanine to prevent membrane anchoring, and then membrane localization. These two mouse models exhibit similar phenotypes in term of reproductive function (ovaries, fertility), except for the uterine response to E2. Indeed, in our C451A-ERα mouse model, the uterine response appears normal at the dose and time tested (0.01 mg/60 days at 24H), while in the NOER mice, the uterus is hypoplasic. The dose used to test the proliferative response in the NOER mice was very high (5mg/60 days) which could have largely impaired the proliferative response since the E2 response in the uterus follows a non-monotonic curve ((Fontaine et al., 2020). Moreover, the E2 uterine response of the same NOER mice used in the Gusfafsson's lab was not so dramatically affected and exhibited a 40 to 70 % decrease of response (Gustafsson et al., 2016). Finally, we totally agree that we only reported ~60-70% reduction in membrane ERα expression in hepatocytes using sucrose gradient. We used this procedure since it was really difficult to visualize it at the plasma membrane by immunofluorescence due to its very low amount of membrane expression (Only 5%) and we can only visualize it in overexpressing conditions (Razandi et al., 2003). The result of the sucrose gradient did not demonstrate 100% loss of ERα expression, but this procedure is quite difficult due to both the very low amount of ERα membrane expression and to the risk of contaminations between sucrose layers that could have slightly altered our final results.

© 2022. Published by The Company of Biologists under the terms of the Creative Commons Attribution License (https://creativecommons.org/licenses/by/4.0/).

For all these reasons, we believe that there is no rationale for considering these two mouse models as different since it is exactly the same mutation that has been introduced. We thus respectfully argue against mentioning this in the introduction, which would lead to more confusion than anything. However, we will highlight the discrepancies in the results obtained with the two mouse lines. This is explained here below.
As requested by the reviewer, we have briefly described the model for membrane loss of functions along with the phenotype of both mouse models even for the uterine response where there is some divergence between the 2 mice but also between authors for NOER mice (Pedram et al., 2014; Gustafsson et al., 2016). This has been introduced from lines 93-106 “These non-genomic pathways activate very rapid signalling (from seconds to few minutes), such as an increase in cAMP in the uterus in response to E2 (Szego and Davis, 1967) and the activation of several kinase cascades (Arnal et al., 2017). Two similar knock-in mouse models selectively inactivated for membrane functions of ERα have been generated by mutating the cysteine 451 into an alanine (447counterpart in humans) that impairs ERα palmitoylation and then membrane localization. Both C451A-ERα (Adlanmerini et al., 2014) and nuclear-only ERα (NOER) mice (Pedram et al., 2014) show abnormal ovaries with hemorrhagic cysts and no corpus luteum suggesting anovulation, while the uterine response to E2 varies from normal in C451A-ERα (Adlanmerini et al., 2014) to a 40 to 100 % reduction in NOER mice (Pedram et al., 2014; Gustafsson et al., 2016). Moreover, rapid vascular effects of E2, such as vasodilatation, acceleration of endothelial repair, and endothelial NO synthase phosphorylation, were abrogated in C451A-ERα mice (Adlanmerini et al., 2014).”

•In the introduction, the essential role of estrogen in embryo implantation via stimulation of LIF release from uterine glands should be acknowledged. Likewise, the discussion should consider whether this role of estrogen is at all responsible for the phenotype of failed implantation sites at 9.5
It was quite difficult to mention this very interesting factor in the introduction. Nevertheless, we have mentioned it on the discussion (lines 462-464) : “Moreover, uterine glands also secrete some other important stromal uterine factors such as LIF (leukaemia inhibitory factor, a member of the interleukin-6 (IL-6) family). LIF is highly induced in response to the nidatory surge in ovarian estrogen at the beginning of pregnancy and is essential in embryo implantation (Stewart et al., 1992; Kelleher et al., 2019). Whether secretion of this factor is altered in C451A-ERa remains an open question.”

•The observation of increased placental weight/area without increased fetal weight, and an increase in the diameter and appearance of vascular channels in the junctional zone suggests a problem with venous drainage from the placenta, causing edema. A few major canals close to the center of the placenta carry maternal blood from the uterine spiral arteries through the junctional zone to the labyrinth, while a larger number of smaller spaces carry blood back from the maternal blood sinuses in the labyrinth to the maternal veins, and these are what appear to be dilated in the placentas from mutant dams. Again, this is kind of fascinating.
We thank the reviewer for this enthusiasm on our results.

Additional minor comments:
Line 182 “certainly due to a progesterone withdrawal failure.” It would be more accurate to say it is likely due to progesterone withdrawal failure.
We agree with the reviewer. This sentence has been modified accordingly: “due to a progesterone withdrawal failure at the end of gestation” was replaced by “likely due to progesterone withdrawal failure” on line 184.
It has also been changed in the discussion, on line 482 where “probably” has been replaced by “likely”.
Although the data clearly demonstrate a failure of progesterone withdrawal, they don’t actually prove that it is solely responsible for the delayed parturition. An experiment showing that progesterone inhibition prevents the delay would be needed for certainty.
We completely agree on this point. Recently, this kind of experiment has been performed on WT mice, and treatment with RU486 (P4-antagonist) at E16 induces pre-term parturition in contrast to progesterone treatment that prevents term parturition (Edey et al., 2018). The suggested experiment (progesterone inhibition) would indeed be necessary to prove that progesterone is solely responsible for the delayed parturition, but it is not the main point of the manuscript. Therefore, we inserted this reference in the discussion to clearly raise this point, on lines 478-480: “This observation is in agreement with the pre-term parturition induced in WT mice treated with

© 2022. Published by The Company of Biologists under the terms of the Creative Commons Attribution License (https://creativecommons.org/licenses/by/4.0/).

the progesterone antagonist RU486 while progesterone treatment delayed parturition (Edey et al., 2018)"

On what basis are mice in figure 1 separated into pregnant/ not-pregnant/ aborted? If both "pregnant" and "aborted" were plug positive, but one gave birth to pups and one didn't, then these don't seem like quite the correct terminology (i.e. aborted is an assumption). If weight is being used to assign categories, then presenting weight data is circular reasoning…
We agree that the term "aborted" is probably not the correct terminology even though resorptions were detected in the uteri of 2 mice sacrificed at E14. Since they could not be considered as pregnant (as we could not determine the presence of embryos. None were found in the females that were sacrificed at E14, and no pups were found in the nest of those which were left undisturbed until parturition) nor non-pregnant (since resorptions were detected in some), the inclusions of these mice either in the non-pregnant group or the pregnant group seems inaccurate. We have thus replaced the terminology "aborted" by "low weight gain" to avoid any mis-interpretation and circular reasoning. We have clearly mentioned this denomination in the materials and methods, the results section and in the figure legend: "Mice were considered as pregnant if they had taken >1g on E7 or as non-pregnant if they had taken <1g on E7. However, if they had taken >1g on E7 but their weight gain had not doubled on E14, mice were classified as low weight gain. Resorptions were detected in the uteri of 2 mice sacrificed in E14 demonstrating that they could not be considered as pregnant nor non-pregnant."

Please use official nomenclature. In particular, ERα is now ESR1.
We have mentioned the nomenclature ESR1 in the text for clarity, in the introduction (ERα (ESR1) and ERß (ESR2), but as the process studied here relates to a post-translational modification we believe it is important to stick with the most common protein name ERα in the rest of the manuscript.

Line 93 I don't think "invalidated" (i.e. disproved) conveys the intended meaning here. Maybe "inactivated"?
We have indeed replaced "invalidated" by "inactivated".

Typo on line 221: Sentence ends with "while"
This word "While" at the end of the sentence has been removed.

References:
1. Adlanmerini, M., Solinhac, R., Abot, A., Fabre, A., Raymond-Letron, I., Guihot, A. L., Boudou, F., Sautier, L., Vessieres, E., Kim, S. H. et al. (2014) 'Mutation of the palmitoylation site of estrogen receptor alpha in vivo reveals tissue-specific roles for membrane versus nuclear actions', Proc Natl Acad Sci U S A 111(2): E283-90.
2. Arnal, J. F., Lenfant, F., Metivier, R., Flouriot, G., Henrion, D., Adlanmerini, M., Fontaine, C., Gourdy, P., Chambon, P., Katzenellenbogen, B. et al. (2017) 'Membrane and Nuclear Estrogen Receptor Alpha Actions: From Tissue Specificity to Medical Implications', Physiol Rev 97(3): 1045-1087.
3. Edey, L. F., Georgiou, H., O'Dea, K. P., Mesiano, S., Herbert, B. R., Lei, K., Hua, R., Markovic, D., Waddington, S. N., MacIntyre, D. et al. (2018) 'Progesterone, the maternal immune system and the onset of parturition in the mouse', Biol Reprod 98(3): 376-395.
4. Fontaine, C., Buscato, M., Vinel, A., Giton, F., Raymond-Letron, I., Kim, S. H., Katzenellenbogen, B. S., Katzenellenbogen, J. A., Gourdy, P., Milon, A. et al. (2020) 'The tissue-specific effects of different 17beta-estradiol doses reveal the key sensitizing role of AF1 domain in ERalpha activity', Mol Cell Endocrinol 505: 110741.
5. Gibson, D. A., Greaves, E., Critchley, H. O. and Saunders, P. T. (2015) 'Estrogen-dependent regulation of human uterine natural killer cells promotes vascular remodelling via secretion of CCL2', Hum Reprod 30(6): 1290-301.
6. Gustafsson, K. L., Farman, H., Henning, P., Lionikaite, V., Moverare-Skrtic, S., Wu, J., Ryberg, H., Koskela, A., Gustafsson, J. A., Tuukkanen, J. et al. (2016) 'The role of membrane ERalpha signaling in bone and other major estrogen responsive tissues', Sci Rep 6: 29473.
7. Kelleher, A. M., DeMayo, F. J. and Spencer, T. E. (2019) 'Uterine Glands: Developmental Biology and Functional Roles in Pregnancy', Endocr Rev 40(5): 1424-1445.

© 2022. Published by The Company of Biologists under the terms of the Creative Commons Attribution License (https://creativecommons.org/licenses/by/4.0/).

8. Moffett, A. and Loke, C. (2006) 'Immunology of placentation in eutherian mammals', Nat Rev Immunol 6(8): 584-94.
9. Pedram, A., Razandi, M., Lewis, M., Hammes, S. and Levin, E. R. (2014) 'Membrane-localized estrogen receptor alpha is required for normal organ development and function', Dev Cell 29(4): 482-90.
10. Razandi, M., Alton, G., Pedram, A., Ghonshani, S., Webb, P. and Levin, E. R. (2003) 'Identification of a structural determinant necessary for the localization and function of estrogen receptor alpha at the plasma membrane', Mol Cell Biol 23(5): 1633-46.
11. Stewart, C. L., Kaspar, P., Brunet, L. J., Bhatt, H., Gadi, I., Kontgen, F. and Abbondanzo, S. J. (1992) 'Blastocyst implantation depends on maternal expression of leukaemia inhibitory factor', Nature 359(6390): 76-9.
12. Szego, C. M. and Davis, J. S. (1967) 'Adenosine 3',5'-monophosphate in rat uterus: acute elevation by estrogen', Proc Natl Acad Sci U S A 58(4): 1711-8.

Second decision letter

MS ID#: DEVELOP/2022/200683

MS TITLE: Loss of function of maternal Membrane Estrogen Receptor ERÎ± alters expansion of trophoblast cells and impacts mouse fertility

AUTHORS: Mariam Rusidze, Melanie Faure, Pierre Sicard, Isabelle Raymond-Letron, Frank GITON, Emilie Vessieres, Vincent PREVOT, Daniel HENRION, Jean-Francois ARNAL, Charlotte Cornil, and Francoise LENFANT
ARTICLE TYPE: Research Article

I am happy to tell you that your manuscript has been accepted for publication in Development, pending our standard ethics checks.

Reviewer 1

Advance summary and potential significance to field

My concerns have been satisfied by the revisions. Please see previous review for summary of advance and signficance to field

Comments for the author

N/A

© 2022. Published by The Company of Biologists under the terms of the Creative Commons Attribution License (https://creativecommons.org/licenses/by/4.0/).
